# Marek’s disease virus Meq oncoprotein interacts with chicken HDAC 1 and 2 and mediates their degradation via proteasome dependent pathway

**DOI:** 10.1038/s41598-020-80792-2

**Published:** 2021-01-12

**Authors:** Yifei Liao, Blanca Lupiani, Yoshihiro Izumiya, Sanjay M. Reddy

**Affiliations:** 1 Department of Veterinary Pathobiology, College of Veterinary Medicine & Biomedical Sciences, Texas A&M University, MS4467, TAMU, College Station, TX 77843 USA; 2 Department of Dermatology, School of Medicine, University of California, Davis, Sacramento, CA USA

**Keywords:** Microbiology, Molecular biology

## Abstract

Marek’s disease virus (MDV) encodes a basic-leucine zipper (BZIP) protein, Meq, which is considered the major MDV oncoprotein. It has been reported that the oncogenicity of Meq is associated with its interaction with C-terminal binding protein 1 (CtBP), which is also an interaction partner of Epstein-Barr virus encoded EBNA3A and EBNA3C oncoproteins. Since both EBNA3C and CtBP interact with histone deacetylase 1 (HDAC1) and HDAC2, we examined whether Meq shares this interaction with chicken HDAC1 (chHDAC1) and chHDAC2. Using confocal microscopy analysis, we show that Meq co-localizes with chHDAC1 and chHDAC2 in the nuclei of MDV lymphoblastoid tumor cells. In addition, immunoprecipitation assays demonstrate that Meq interacts with chHDAC1 and chHDAC2 in transfected cells and MDV lymphoblastoid tumor cells. Using deletion mutants, interaction domains were mapped to the N-terminal dimerization domain of chHDAC1 and chHDAC2, and the BZIP domain of Meq. Our results further demonstrate that this interaction mediates the degradation of chHDAC1 and chHDAC2 via the proteasome dependent pathway. In addition, our results show that Meq also induces the reduction of global ubiquitinated proteins through a proteasome dependent pathway. In conclusion, our results provide evidence that Meq interacts with chHDAC1 and chHDAC2, and induces their proteasome dependent degradation.

## Introduction

Marek’s disease (MD) is a highly contagious lymphoproliferative disease of chicken caused by an avian alphaherpesvirus, Marek’s disease virus (MDV). Infection with MDV results in paralysis, neurological disease, T-cell lymphomas, and immunosuppression in infected chickens^1^. Due to occasional outbreaks and the need of more than five billion doses of MDV vaccine annually, MD is still an economically significant disease for the poultry industry^2^. The genome of MDV consists of two unique regions flanked by inverted repeat regions; proteins and RNAs (such as Meq, viral telomerase RNA and microRNAs) that are highly expressed in MDV lymphoblastoid tumor cells, are encoded within the repeat regions^3–5^. *meq* is consistently expressed both during the lytic phase and in lymphoblastoid tumor cells^6^. Meq has been shown to be essential for MDV induced transformation of T lymphocytes, but dispensable for lytic infection^3^.

MDV Meq is a 339 amino acid long protein, encoded in the MDV EcoRI Q fragment of the MDV-1 genome^6^. Meq consists of an N-terminal basic region (BR) and a leucine zipper (ZIP) domain, as well as a C-terminal transcriptional regulatory domain^7^. The basic-leucine zipper (BZIP) domain of Meq shares significant homology with the Jun/Fos family of transcription factors and also forms heterodimers with Jun/Fos as well as homodimers with itself^8^. Using antisense RNA that specifically targets the *meq* gene, Xie et al. demonstrated the importance of Meq in maintaining the transformed status of MSB-1, an MDV transformed lymphoblastoid cell line^9^. Without an optimal in vitro chicken T cell transformation system, the direct transformation properties of Meq were first characterized in a rodent fibroblast (Rat-2) cell line^10^. Ectopic expression of Meq resulted in transformation of Rat-2 cells characterized by anchorage- and serum- independent growth as well as morphological transformation, and resistance to apoptosis^10^. Lupiani et al. showed that infection with a Meq null virus did not induce MD associated lymphomas in infected chickens, even though the virus replicated robustly during early cytolytic phase, providing the first conclusive evidence that Meq plays an essential role in transformation of lymphocytes^3^. Later, Levy et al*.* revealed that Meq induced transformation of DF-1 cells, an immortalized chicken embryo fibroblast cell line, is through a v-Jun pathway^11^. In addition to transformation, Meq has been shown to interact with multiple cellular proteins, regulate cellular signaling pathways, and bind to both viral and host genomes^12^. The interaction of Meq and c-Jun has been well studied, and Meq-Jun heterodimers bind to AP-1 sequence to transactivate target gene expression^7^. Some other AP-1 transcription factors, including Fos, CREB, and ATF family members, also interact with Meq^13^. It has been shown that Meq interacts with p53 tumor suppressor protein and suppresses p53 mediated apoptosis and transcriptional regulation^14^. In addition, the interaction between Meq and C-terminal binding protein 1 (CtBP) has been demonstrated to be critical for Meq induced T cell lymphomas^15^.

Recently, post-translational modifications of histones have been identified as critical regulatory factors of viral gene expression during herpesvirus infection, which is one of the mechanisms that host cells utilize as anti-viral response towards incoming herpesvirus genomes^16^. As a consequence, herpesvirus have developed mechanisms to manipulate and interfere with histone-modifying enzymes to benefit their replication and gene expression in host cells. Acetylation is one of the most well studied modifications of histone proteins, a reversible modification that occurs on lysine (K) residues. There are numerous histone acetyltransferases (HATs) and their activity can be reversed by the activity of histone deacetylases (HDACs). In mammals, eighteen HDACs have been identified and are classified into four different groups. Class I HDACs, including HDAC1, 2, 3 and 8, are the most studied. Especially, HDAC1 and HDAC2 (HDAC1 and 2) have been showed to be involved in the formation of at least three distinct repressor complexes, including Sin3, CoREST and NuRD^17^. Post-translational modifications, including phosphorylation, ubiquitination, and SUMOylation, of HDAC1 and 2 have also been extensively studied^18^. Phosphorylation of HDAC1 and 2 regulates their transcriptional regulation activity, enzymatic activity, and protein interactions^18^. Casein kinase II (CKII) has been identified as the main cellular upstream protein kinase responsible for phosphorylation of HDAC1 and 2 in vivo^19^. Early studies have shown that HDAC1 and 2 are also phosphorylated by alphaherpesvirus encoded U_S_3 serine/threonine protein kinase^20,21^. Ubiquitin and small ubiquitin-like modifier (SUMO) are small regulatory proteins which share a similar modification machinery, mediated by three enzymes: E1 activating enzyme, E2 conjugating enzyme and E3 ligase. Covalent attachment of ubiquitin targets proteins for degradation through the proteasome pathway, while modification by SUMO usually regulates the target protein activity and cellular localization^22,23^. Both HDAC1 and HDAC2 have been reported to be ubiquitinated and degraded by the proteasome dependent pathway^18^. Ubiquitination and degradation of HDAC1 has been shown to correlate with the enhanced metastatic activity of prostate and breast cancer cell lines^24^. However, even though both HDAC1 and HDAC2 have been shown to be SUMOylated, the biological significances of their SUMOylation are still under investigation^18^.

The interaction between *alpha*, *beta*, and *gamma* herpesviruses with HDACs has been widely studied, and exhibits different effects. Treatment of herpesvirus latently infected cells with HDAC inhibitors (HDACi), reactivates virus and dramatically remodels viral genome architecture indicating HDACs play an important role in regulating herpesvirus latency^17,25^. Upon infection with herpes simplex virus 1 (HSV-1), viral protein ICP8 translocates HDAC1/CoREST/LSD1 to the cytoplasm, and ICP0 interacts with HDAC1 to disrupt the CoREST repressor complex and translocates HDAC1 to ND10 bodies^26^. It has been shown that human cytomegalovirus (HCMV) pUL28/29 and pUL38 proteins cooperate with NuRD complex to promote the expression of immediate-early genes during infection^27^. Epstein–Barr virus (EBV) nuclear antigen 3C (EBNA3C) interacts with HDAC1 and 2 to repress viral gene expression and promote the association between HDAC1 and CBF1/RPB-Jk^28^. In addition, like MDV, EBV and Kaposi's sarcoma-associated herpesvirus (KSHV) encode a BZIP protein, BZLF1 and K-bZIP, respectively, and it has been reported that the SUMOylated BZLF1 interacts with and recruits HDAC3 to BZLF1 responsive promoters to repress their transcriptional activity^29^; on the other hand, K-bZIP interacts with HDAC2 via the leucine zipper domain and recruits it to the promoters of OriLyt and ORF50^30^. Even though HDACs play an important role in herpesvirus gene regulation, there are no studies on MDV proteins-HDACs interactions. Here, we investigate the existence and implications of Meq-HDAC interactions.

In this study, we show that Meq interacts with chicken HDAC1 and HDAC2 (chHDAC1 and 2) at the N-terminal dimerization domain of chHDAC1 and 2, and that Meq mediates the degradation of chHDAC1 and 2 via the proteasome dependent pathway. We also identified that the N-terminal, mainly the BZIP domain, of Meq is important for its association with chHDAC1 and 2. In addition, our results demonstrate that the N-terminal region of Meq is also important for Meq mediated reduction of global ubiquitinated proteins. In conclusion, our results illustrate that MDV Meq functionally interacts with chHDAC1 and 2, leading to their degradation through the proteasome dependent pathway.

## Results

### MDV Meq co-localizes and interacts with chHDAC1 and 2

**Figure 1:**
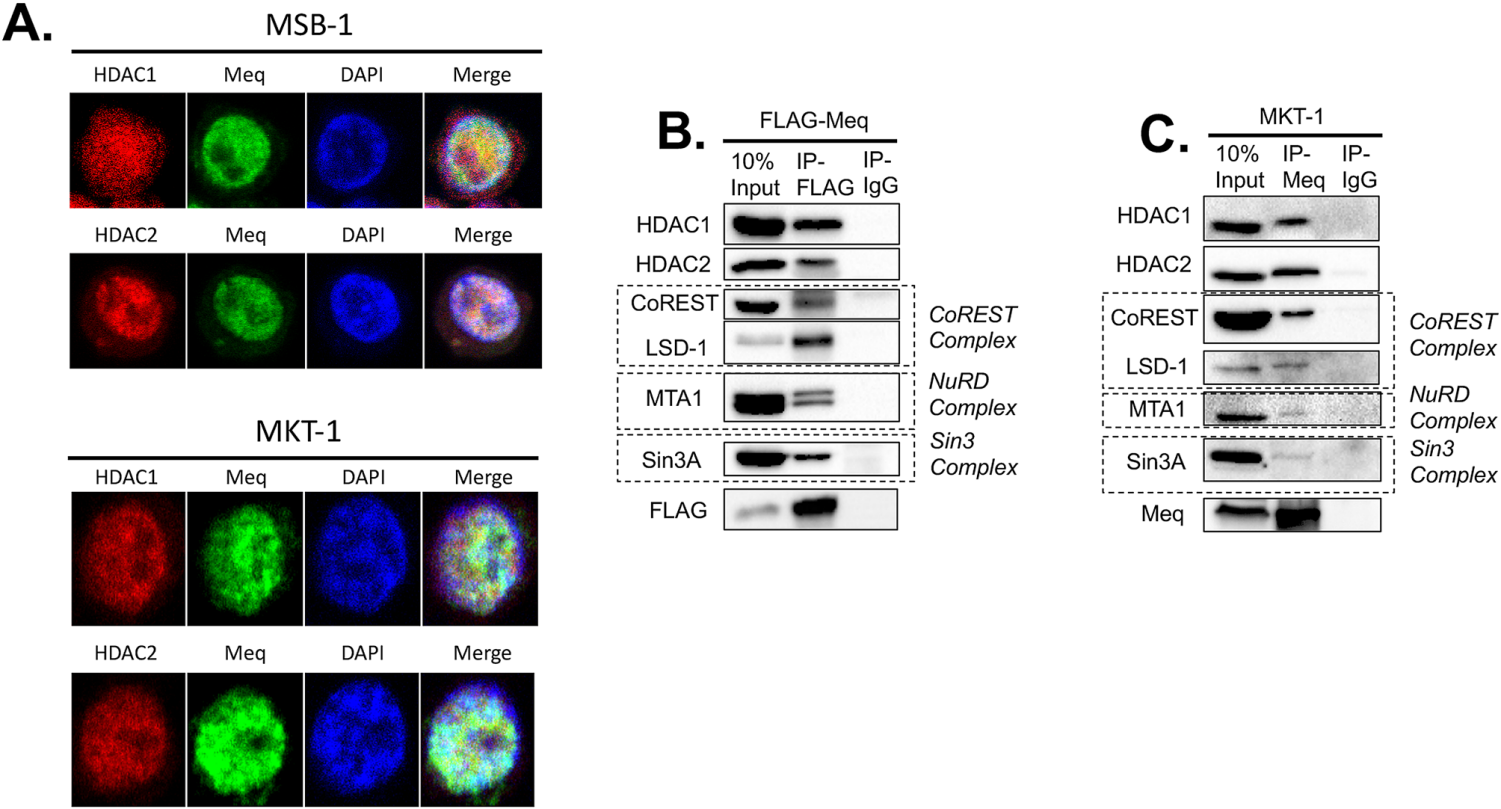
MDV Meq co-localizes and interacts with chHDAC1 and 2. (**A**) MSB-1 and MKT-1 tumor cells were fixed and incubated with mouse anti-HDAC1 or mouse anti-HDAC2 and rabbit anti-Meq antibodies, followed by goat anti-mouse-Texas Red and goat anti-rabbit-Alex Flour 488. DAPI was used to stain cell nuclei. All images were taken by confocal microscopy. (**B**) 293T cells were transfected with pcDNA-FLAG-Meq. Forty-eight hours later, whole cell lysates were subjected to immunoprecipitation (IP) with FLAG antibody and normal mouse IgG. Western blot (WB) analysis was performed with antibodies against HDAC1, HDAC2 and components of the CoREST, NuRD, and Sin3 protein complexes. (**C**) MKT-1 tumor cells were lysed and subjected to IP with anti-Meq polyclonal antibody and normal rabbit IgG, followed by WB with the indicated antibodies.

To explore the potential association between MDV Meq with chHDAC1 and 2, we first examined the subcellular localization of Meq and chHDAC1 and 2 in MDV lymphoblastoid tumor cells. We performed immunofluorescence assay (IFA) with two MDV lymphoblastoid tumor cell lines, MSB-1 and MKT-1, using antibodies against Meq and HDAC1 or HDAC2. As shown in Fig. 1A, the majority of MDV Meq (green) and chHDAC1 and 2 (red) are distribute throughout the cell nucleus (blue) of MDV lymphoblastoid tumor cells, and the merged images suggest Meq may co-localize with chHDAC1 and 2. As stated in the introduction, HDAC1 and 2 are components of CoREST, NuRD and Sin3 protein complexes^18^. We further studied the interaction between MDV Meq and HDAC1 and 2, as well as other protein components of these protein complexes. Immunoprecipitation (IP) assays with whole cell lysates of pcDNA-FLAG-Meq transfected 293T cells and MDV lymphoblastoid tumor cell line show that Meq could efficiently co-precipitate HDAC1 and 2, as well as protein components of the CoREST (CoREST and LSD-1), NuRD (MTA-1), and Sin3 (Sin3A) complexes in 293T cells (Fig. 1B and Supplementary Fig. S2 online) and MDV lymphoblastoid tumor cells (MKT-1) (Fig. 1C and Supplementary Fig. S2 online). We also performed reciprocal pull-down experiments with MKT-1 cell lysates, and the results show that chHDAC1 and 2 can efficiently co-precipitate Meq (Supplementary Fig. S1A, S1B and S1D, S1E online). The interactions between Meq and chHDAC1 and 2 were also observed in MDV infected chicken embryonic fibroblasts (data not shown).

It has been shown that Meq represses the transcriptional activity of an MDV bi-directional promoter, which regulates the expression of MDV *pp38* and *pp14*^13^. Here, we characterized the potential function of Meq and chHDAC1 and 2 interactions in the transcriptional regulation of these promoters. As shown in Supplementary Fig. S1C online, we found that Meq and chHDAC2 independently downregulate the transcriptional activity of both *pp14* and *pp38* promoters, while chHDAC1 only represses *pp14* promoter activity. However, no significant cooperative function was observed between Meq and chHDAC1 & 2 as co-transfection of Meq with chHDAC1 or chHDAC2 did not affect their individual effect on the transcriptional activity of *pp14* and *pp38* promoters (Supplementary Fig. S1C online).

### The N-terminal dimerization domains of chHDAC1 and 2 mediate the interaction with Meq

**Figure 2:**
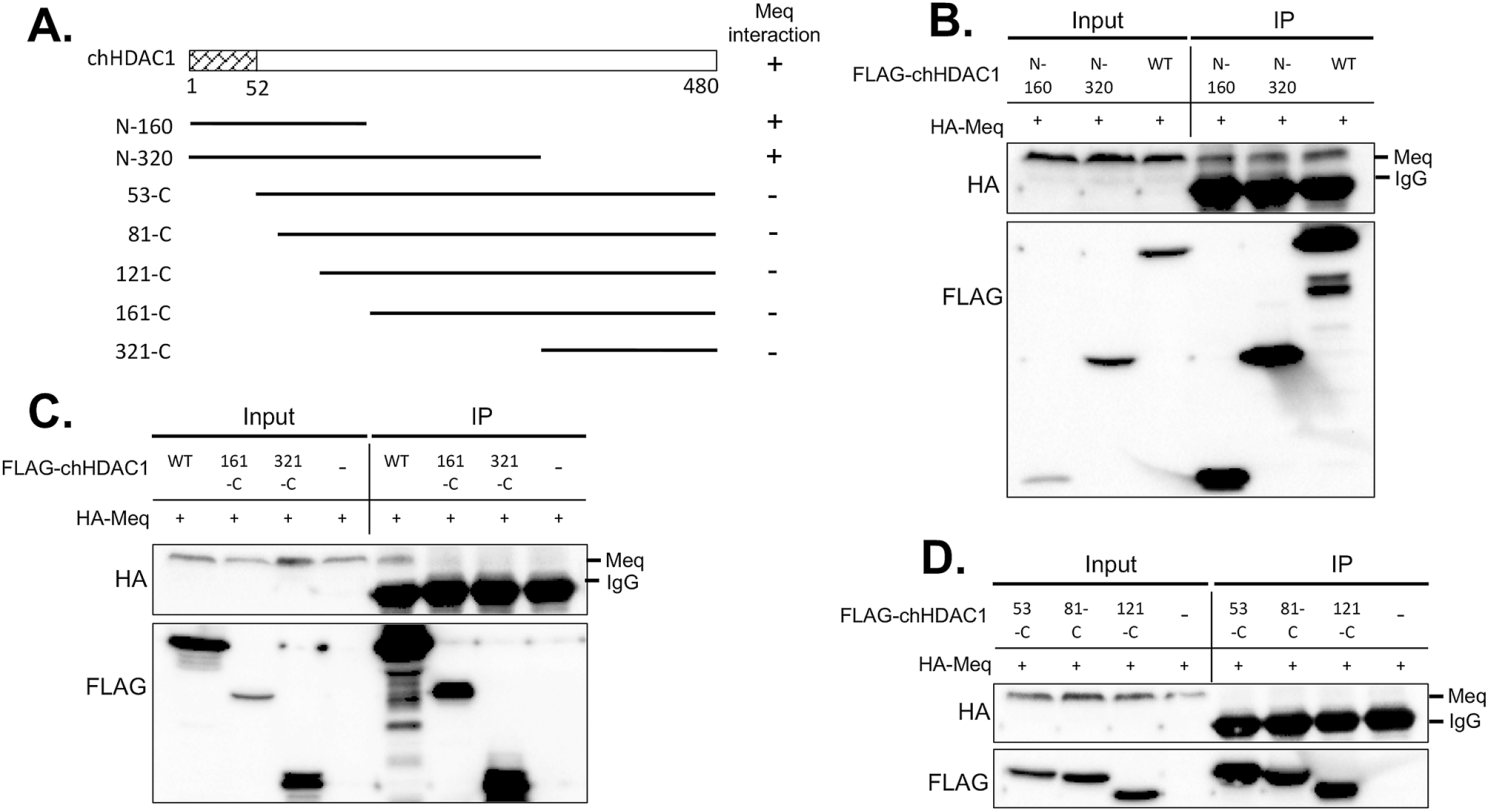
Mapping the domain of chHDAC1 that mediates its interaction with MDV Meq. (**A**) Schematic representation of FLAG tagged pcDNA-chHDAC1 deletion mutants. The N-terminal 52 amino acids were marked as homodimerization domain and Meq interaction domain. The interaction of each chHDAC1 mutant with Meq is indicated on the right: “+” indicates interaction, “−” indicates no interaction. (**B**, **C**, **D**) FLAG tagged pcDNA-chHDAC1 deletion mutants were co-transfected with pcDNA-HA-Meq into 293T cells. Cells were lysed 48 h post transfection and subjected to immunoprecipitation with mouse anti-FLAG agarose beads. Western blot was processed with HA and FLAG antibodies.

**Figure 3:**
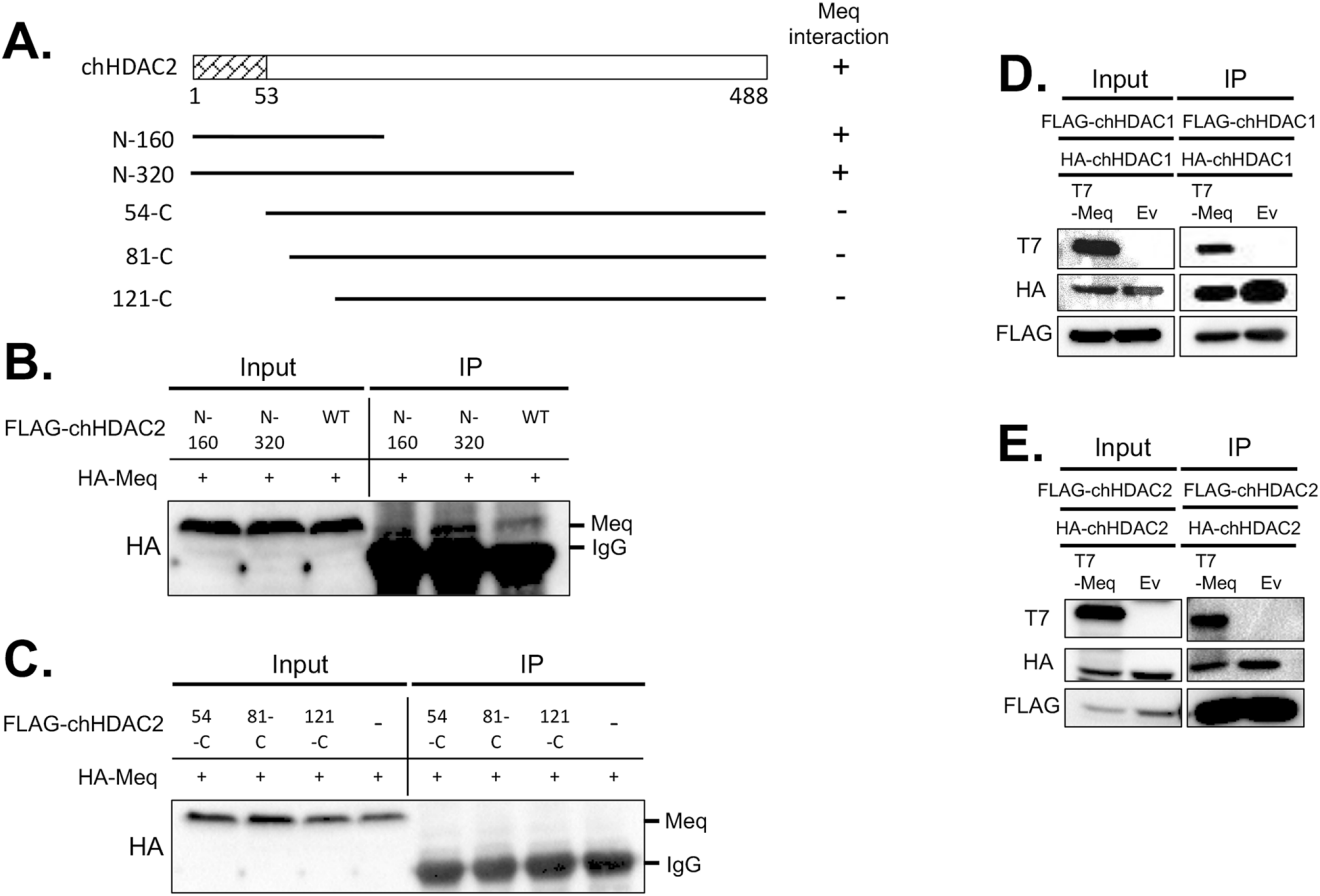
Mapping the domain of chHDAC2 that mediates its interaction with MDV Meq. (**A**) Schematic representation of FLAG tagged pcDNA-chHDAC2 deletion mutants. The N terminal 53 amino acids were marked as homodimerization domain and Meq interaction domain. The interaction of each chHDAC2 mutant with Meq is indicated on the right: “+” indicates interaction, “−” indicates no interaction. (**B**, **C**) pcDNA-FLAG-chHDAC2 deletion mutants were co-transfected with pcDNA-HA-Meq into 293T cells for 48 h. Immunoprecipitation (IP) was performed with mouse anti-FLAG agarose beads, followed by Western blot (WB) analysis with HA antibody. pcDNA-T7-Meq or pcDNA empty vector (Ev) were co-transfected with pcDNA-FLAG-chHDAC1 and pcDNA-HA-chHDAC1 (**D**) or pcDNA-FLAG-chHDAC2 and pcDNA-HA-chHDAC2 (**E**) into 293T cells. Forty-eight hours later, IP was processed with FLAG antibody and normal mouse IgG, followed by WB with T7, HA, and FLAG antibodies.

After demonstrating the interaction between Meq and chHDAC1 and 2, we investigated the domains of chHDAC1 and 2 responsible for these interactions. We first generated several FLAG tagged N-terminal or C-terminal deletion mutants of chHDAC1 in pcDNA (Fig. 2A). These mutants were cotransfected with pcDNA-HA-Meq into 293T cells, followed by IP. Our results show that both N-terminal, N-160 and N-320, of chHDAC1 interact with Meq at levels similar to wild type (WT) chHDAC1 (Fig. 2B and Supplementary Fig. S3 online), while the C-terminal, 161-C and 321-C, constructs did not interact with Meq (Fig. 2C and Supplementary Fig. S3 online), indicating that the N-terminal 160 amino acids of chHDAC1 are enough to mediate its association with Meq. We further shorten the essential interaction domain to the N-terminal 52 amino acids since constructs 53-C, 81-C, and 121-C of chHDAC1 failed to interact with Meq (Fig. 2D and Supplementary Fig. S3 online). Similar results were observed for chHDAC2 with the N-terminal 53 amino acids being required for its interaction with Meq (Fig. 3A–C and Supplementary Fig. S4 online).

Since the N-terminal 52 and 53 amino acids of HDAC1 and HDAC2, respectively, were shown to be their homodimerization domains^31^, we next examined whether the presence of Meq would affect their homodimerization. Plasmids expressing FLAG-chHDAC1 were cotransfected with HA-chHDAC1 in the presence of T7-Meq or empty vector (Ev) into 293T cells. Immunoprecipitation studies showed that the interaction between FLAG-chHDAC1 and HA-chHDAC1 was not affected by the presence of Meq (Fig. 3D and Supplementary Fig. S4 online). Interestingly, the levels of chHDAC1 were lower in the presence of Meq, in both input and IP results, due to Meq mediated degradation of chHDAC1 as shown in the following section. Similarly, Meq did not interfere with the homodimerization of chHDAC2 (Fig. 3E and Supplementary Fig. S4 online).

### The BZIP domain of Meq is important for its interaction with chHDAC1 and 2

**Figure 4:**
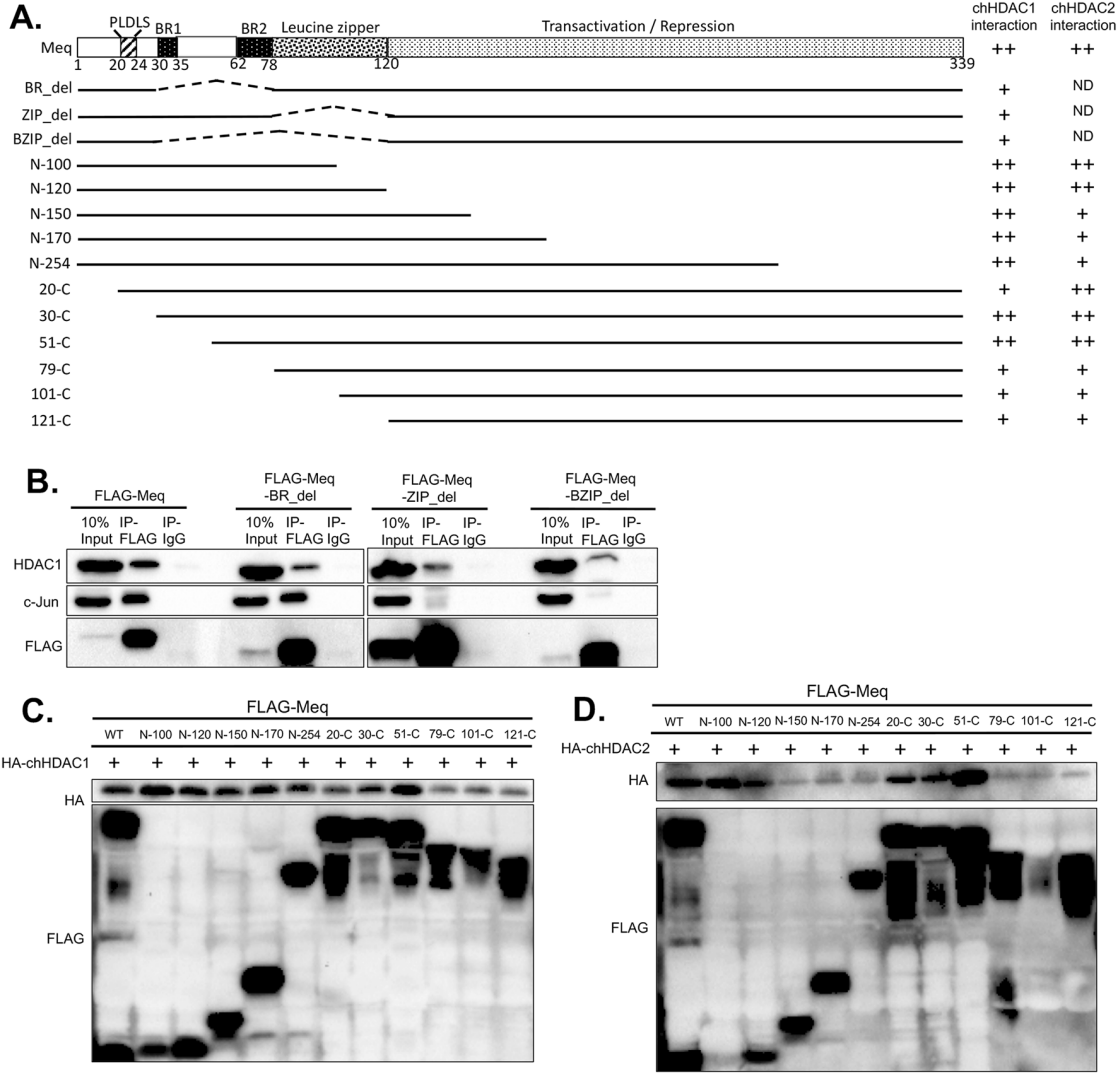
Mapping the domain of Meq that mediates its interaction with chHDAC1 and 2. (**A**) Schematic representation of Meq domains and FLAG tagged pcDNA-Meq deletion mutants. The interaction of each Meq mutant with chHDAC1 and 2 is indicated on the right: “++” indicates strong interaction, “+” indicates weak interaction, “ND” indicates not determined. (**B**) pcDNA-FLAG-Meq deletion mutants were transfected into 293T cells for 48 h. Whole cell lysates were subjected to immunoprecipitation (IP) with rabbit anti-FLAG antibody or normal rabbit IgG, followed by Western blot (WB) analysis with HDAC1, c-Jun, and FLAG antibodies. pcDNA-Meq deletion mutants were co-transfected with pcDNA-HA-chHDAC1 (**C**) or pcDNA-HA-chHDAC2 (**D**) into 293T cells. Whole cell lysates were harvested 48 h post transfection and subjected to IP with mouse anti-FLAG agarose beads. WB was processed with HA and FLAG antibodies.

To identify the domain/s of Meq involved in its interaction with chHDAC1 and 2, we constructed a series of FLAG tagged pcDNA-Meq deletion mutants (Fig. 4A). First, FLAG tagged Meq basic region deletion (BR_del), leucine zipper deletion (ZIP_del), and double deletion (BZIP_del) mutants were cotransfected into 293T cells. Our IP results show that Meq-BR_del, Meq-ZIP_del, and Meq-BZIP_del mutants only weakly interact with chHDAC1 compared to wild type Meq (Fig. 4B and Supplementary Fig. S5 online), indicating that the BZIP region of Meq is important for its association with chHDAC1. We also included c-Jun as a control, since it has been shown to interact with Meq at the ZIP region^32^. Our IP results confirmed that Meq BR deletion mutant, but not Meq ZIP deletion and BZIP deletion mutants, could interact with c-Jun (Fig. 4B and Supplementary Fig. S5 online).

To further explore the interaction region, additional Meq deletion mutants were examined in transfected 293T cells (Fig. 4A). Our IP results show that N-100 of Meq is enough to interact with chHDAC1 at levels similar to wild type Meq (Fig. 4C and Supplementary Fig. S5 online). Furthermore, 51-C of Meq strongly associates with chHDAC1, but 20-C, 79-C, 101-C and 121-C of Meq only weakly interacts with chHDAC1 (Fig. 4C and Supplementary Fig. S5 online), indicating that the N-terminal 20–50 amino acid (including the ‘PLDLS’ CtBP binding motif and BR1 domain) of Meq are not important for its association with chHDAC1. Overall, Fig. 4C and Supplementary Fig. S5 online suggest that amino acids 1–19 and 51–100 (within the BZIP region) of Meq is important for its interaction with chHDAC1. Similarly, the same region, amino acids 51–100, of Meq is also important for its interaction with chHDAC2 (Fig. 4D and Supplementary Fig. S5 online). However, we also observed that although both N-100 and N-120 regions of Meq strongly interact with chHDAC2, N-150, N-170 and N-254 only weakly interact with chHDAC2 (Fig. 4D and Supplementary Fig. S5 online), indicating that amino acids 121–150 of Meq may partially inhibit the interaction between Meq and chHDAC2.

### MDV Meq mediates the degradation of chHDAC1 and 2

**Figure 5:**
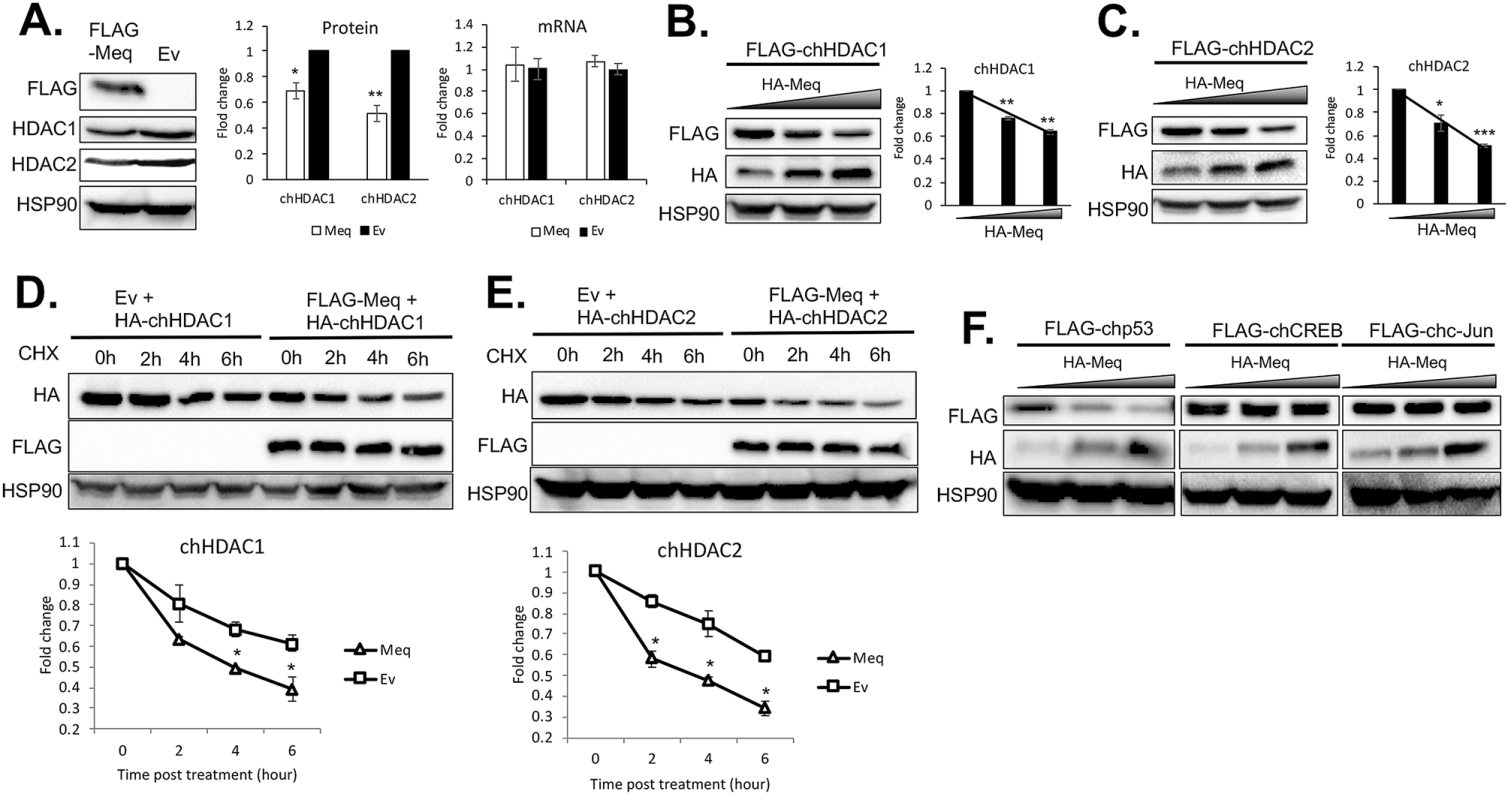
Meq mediates the degradation of chHDAC1 and 2. (**A**) DF-1 cells were transfected with pcDNA-FLAG-Meq or pcDNA empty vector (Ev) and 48 h later, cells were harvested for protein and RNA extraction. Western blot (WB) analysis was processed with the indicated antibodies (left) and quantified with Image J and presented as fold change compared to Ev (middle). qRT-PCR was processed with primers targeting chHDAC1 and chHDAC2 and presented as fold change compared to Ev (right). pcDNA-FLAG-chHDAC1 (**B**) or pcDNA-FLAG-chHDAC2 (**C**) were co-transfected with different amounts of pcDNA-HA-Meq into 293T cells for 48 h. Whole cell lysates were subjected to WB with the indicated antibodies (left). WB results were quantified with Image J, normalized to HSP90, and presented as fold change compared to the least amount of Meq transfection (right). pcDNA-FLAG-Meq or pcDNA Ev were co-transfected with pcDNA-HA-chHDAC1 (**D**) or pcDNA-HA-chHDAC2 (E) into 293T cells and 24 h later, cells were treated with cycloheximide (CHX, 1 mg/ml) for the indicated length of time. WB were performed with HA, FLAG, and HSP90 antibodies (upper). HA-chHDAC1 or HA-chHDAC2 protein levels were quantified with Image J, normalized to HSP90, and presented as fold change compared to non-treated cells (bottom). All experiments were repeated two times. Error bars indicate standard deviation (SD). (**F**) pcDNA-FLAG-chp53, pcDNA-FLAG-chCREB, or pcDNA-FLAG-chc-Jun were co-transfected with different amounts of pcDNA-HA-Meq into 293T cells and 48 h later, whole cell lysates were subjected to WB with the indicated antibodies. The statistical differences were analyzed by Student *t* test. *p < 0.05, **p < 0.01, ***p < 0.001.

During the course of our studies, apart from interaction, we observed that levels of chHDAC1 and 2 proteins were lower in the presence of Meq (Fig. 3D, E, and Supplementary Fig. S4 online) in transfected 293T cells. We further confirmed our results in DF-1 cells transfected with pcDNA-FLAG-Meq. As shown in Fig. 5A left and Supplementary Fig. S6 online, levels of endogenous chHDAC1 and 2 were reduced by ~ 1.4 and 1.9 fold, respectively, in the presence of Meq and this reduced levels of chHDAC1 and 2 by Meq were not regulated at the transcriptional level as only the protein levels (Fig. 5A middle, Protein), but not mRNA levels (Fig. 5A right, mRNA), of chHDAC1 and 2 were affected by the presence of Meq. Furthermore, our results show that chHDAC1 (Fig. 5B and Supplementary Fig. S6 online) or chHDAC2 (Fig. 5C and Supplementary Fig. S6 online) levels were reduced in a Meq dose dependent manner.

Next, we examined the stability of chHDAC1 and 2 in cells co-transfected with pcDNA-FLAG-Meq or pcDNA empty vector (Ev), in the presence of cycloheximide (CHX), a protein synthesis inhibitor. Compared to Ev, levels of chHDAC1 were significantly lower in the presence of Meq at 4- and 6-h post CHX treatment (Fig. 5D and Supplementary Fig. S6 online). Similarly, levels of chHDAC2 were significantly lower in the presence of Meq at all time points post CHX treatment (Fig. 5E and Supplementary Fig. S6 online). In addition, we examined the effect of Meq on levels of other interaction partners, including p53, CREB, and c-Jun^13,14,33^. Our results show that the levels of chicken p53 (chp53), but not chCREB and chc-Jun, were reduced by increasing amounts of Meq protein (Fig. 5F and Supplementary Fig. S6 online), indicating that Meq does not mediate the degradation of all interaction partners. Overall, these results suggest that MDV Meq could partially mediate the degradation of endogenous and exogenous chHDAC1 and 2.

### MDV Meq mediates the degradation of chHDAC1 and 2 via the proteasome dependent pathway

**Figure 6:**
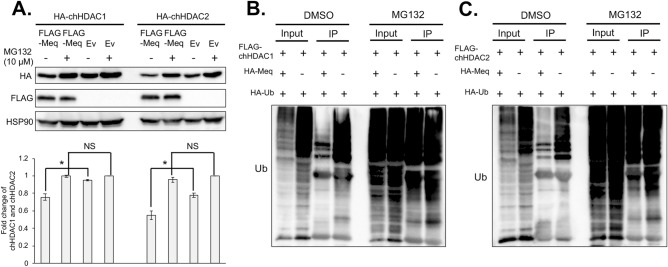
MDV Meq mediates the partial degradation of chHDAC1 and 2 via the proteasome dependent pathway. (**A**) pcDNA-HA-chHDAC1 or pcDNA-HA-chHDAC2 were co-transfected with pcDNA-FLAG-Meq or pcDNA empty vector (Ev) into 293T cells and 24 h later, cells were treated overnight with or without MG132 (10 μM). Western blot (WB) analysis was performed with whole cell lysates using the indicated antibodies. Representative WB images were shown (upper). Protein levels of HA-chHDAC1 or HA-chHDAC2 were quantified with Image J, normalized to HSP90, and presented as fold change compared to MG132 treated pcDNA-HA-chHDAC1 or pcDNA-HA-chHDAC2 and pcDNA Ev cotransfected cells (bottom). The statistical differences were analyzed by Student *t* test. *p < 0.05, *NS* not significant. pcDNA-FLAG-chHDAC1 (**B**) or pcDNA-FLAG-chHDAC2 (**C**) were co-transfected with pcDNA-HA-Meq or pcDNA Ev and pcDNA-HA-Ub into 293T cells. Twenty-four hours post transfection, cells were treated with MG132 (10 μM) or DMSO overnight. Immunoprecipitations were performed with mouse anti-FLAG agarose beads, followed by WB with ubiquitin antibody.

To determine the mechanism responsible for Meq mediated partial degradation of chHDAC1 and 2, we used a proteasome inhibitor, MG132, to treat 293T cells co-transfected with Meq and chHDAC1 or chHDAC2. As shown in Fig. 6A and Supplementary Fig. S7 online, without MG132 treatment, levels of HA-chHDAC1 or HA-chHDAC2 were significant lower in the presence of FLAG-Meq compared to empty vector (Ev) transfected cells; with MG132 (10 μM) treatment, levels of HA-chHDAC1 or HA-chHDAC2 were no different between FLAG-Meq and empty vector (Ev) transfected cells. These results demonstrate that MG132 treatment could inhibit chHDAC1 and 2 degradation, indicating that Meq mediates partial degradation of chHDAC1 and 2 via a proteasome dependent pathway. We further confirmed our results by treating transfected cells with different concentration of MG132 and the results show that treatment with 5 μM MG132 is sufficient to efficiently inhibit Meq mediated degradation of chHDAC1 and 2 (Supplementary Fig. S8A, S8B and S9 online). In addition, MG132 (10 μM) treatment also rescued the degradation of chHDAC1 and 2 in the presence of Meq and CHX (1 mg/ml) (Supplementary Fig. S8C and Fig. S9 online).

Since chHDAC1 and 2 appears to be degraded by a proteasome dependent pathway, a mechanism normally initialized by ubiquitination, we next investigated the ubiquitination of chHDAC1 and 2 in the presence or absence of Meq. 293T cells were co-transfected with pcDNA-FLAG-chHDAC1 or pcDNA-FLAG-chHDAC2 and pcDNA HA tagged ubiquitin (HA-Ub) in the presence or absence of pcDNA-HA-Meq. Twenty-four hours later, cells were treated overnight with DMSO or MG132 and subjected to IP using mouse anti-FLAG agarose beads to precipitate FLAG tagged chHDAC1 and chHDAC2, followed by Western blot analysis with ubiquitin antibody. Our results show that chHDAC1 and 2 were ubiquitinated resulting in higher molecular weight protein species (Fig. 6B, C). In addition, ubiquitinated chHDAC1 and 2 were degraded in the presence of Meq (Fig. 6B, C, DMSO treatment), which can be inhibited by treatment with MG132 (Fig. 6B, C, MG132 treatment). In conclusion, the above results suggest that Meq utilizes a proteasome dependent pathway to induce the partial degradation of chHDAC1 and 2.

### MDV Meq mediates the reduction of global ubiquitinated proteins via a proteasome dependent pathway

**Figure 7:**
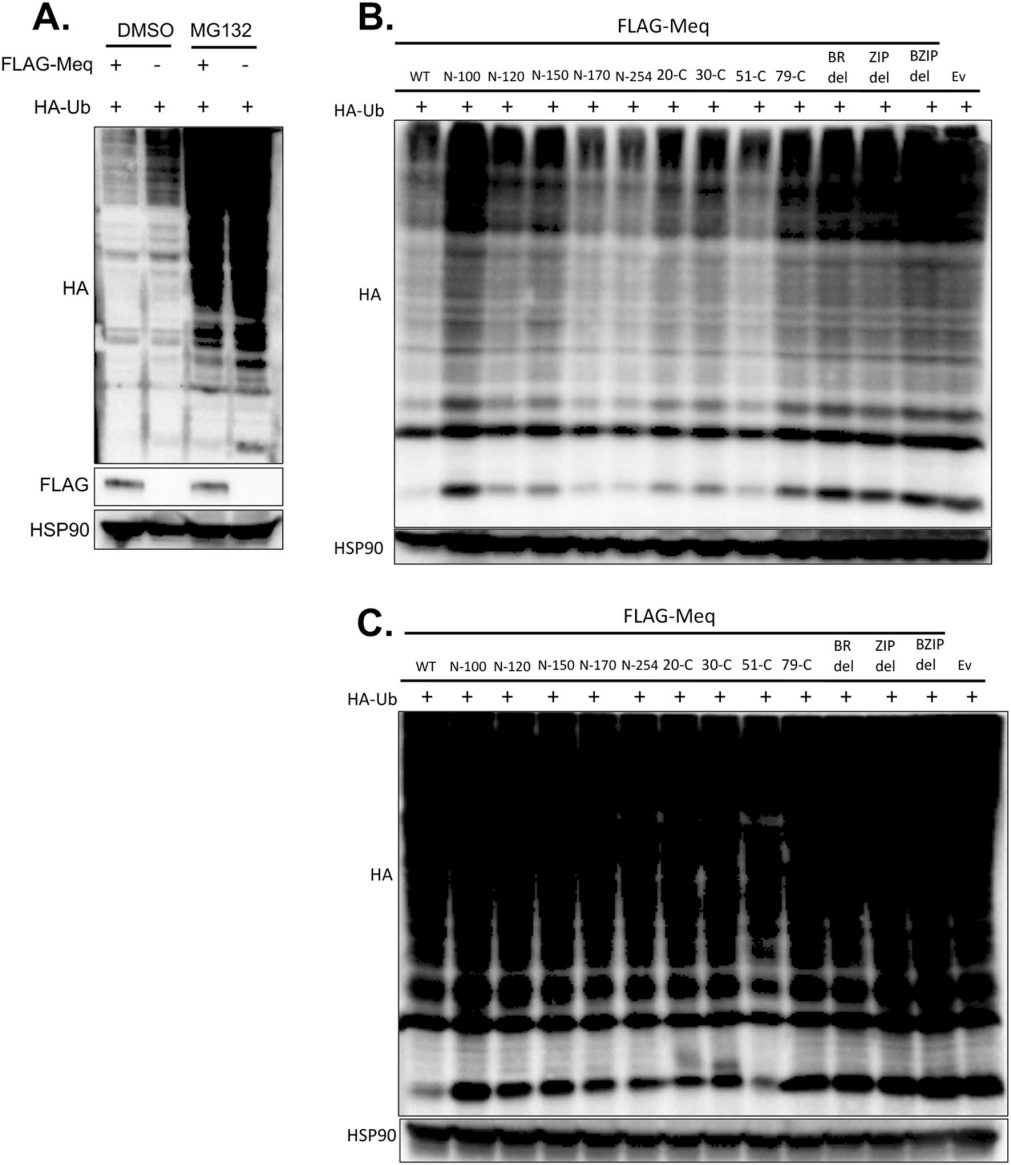
MDV Meq mediates the degradation of global ubiquitinated proteins via the proteasome dependent pathway. (**A**) pcDNA-FLAG-Meq or pcDNA empty vector (Ev) were co-transfected with pcDNA-HA-Ub into 293T cells. Twenty-four hours later, cells were treated with DMSO or MG132 (10 μM) overnight. Cells were lysed and subjected to Western blot (WB) analysis with HA, FLAG, and HSP90 antibodies. (**B**) pcDNA-HA-Ub was cotransfected with the indicated pcDNA-FLAG-Meq deletion mutants or pcDNA Ev into 293T cells and 48 h later, cells were lysed and subjected to WB with HA and HSP90 antibodies. (**C**) pcDNA-HA-Ub was co-transfected with the indicated pcDNA-FLAG-Meq deletion mutants or pcDNA Ev to 293T cells. Twenty-four hours later, cells were treated with MG132 (10 μM) overnight. Cells were lysed and subjected to WB with HA and HSP90 antibodies.

Apart from chHDAC1 and 2, we found that MDV Meq could reduce ubiquitinated protein globally in a proteasome dependent pathway as MG132 treatment restored high molecular weight ubiquitinated proteins in Meq expressing cells (Fig. 7A and Supplementary Fig. S10 online). We further mapped the domain of Meq important for its ability to mediate the reduction of ubiquitinated proteins. We cotransfected the indicated pcDNA-FLAG-Meq deletion mutants and pcDNA-HA-Ub into 293T cells. Western blot results show that N-120 to N-254 of Meq, but not N-100 of Meq, reduce levels of global ubiquitinated proteins (Fig. 7B and Supplementary Fig. S10 online). In addition, we observed that 20-C to 51-C of Meq, but not 79-C, BR_del, ZIP_del and BZIP_del of Meq, reduce the levels of global ubiquitinated proteins (Fig. 7B and Supplementary Fig. S10 online). We further showed that treatment with MG132 could rescue Meq deletion mutants mediated reduction of global ubiquitinated proteins (Fig. 7C and Supplementary Fig. S10 online), indicating this process is also a proteasome dependent pathway. Taken together, we concluded that amino acids 51–120, which are within the BZIP region, are important for Meq mediated reduction of global ubiquitinated proteins.

## Discussion

Meq is a oncoprotein encoded by MDV, which plays an essential role in MDV induced transformation of T lymphocytes in chickens^3^. Apart from its transformation property, multiple functions have been attributed to Meq, including transcription regulation, apoptosis inhibition, protein interactions and DNA binding^11,13–15,34^. However, the role of many Meq-host interactions are still not fully understood. It has been shown that the oncogenic property of Meq are related to its interaction with CtBP, which also interacts with adenovirus encoded E1A protein and EBV encoded EBNA3A and EBNA3C proteins^15^.

HDACs are a class of modification enzymes that can remove acetyl molecules from lysine ε-NH_3_ groups. In total, 18 HDACs have been identified so far in humans and have been classified into four classes. Numerous studies emphasize the critical role of histone and non-histone proteins acetylation in herpesviruses infection^16,35^. HDACs are prominent regulators of protein acetylation and play essential roles in gene expression regulation, thus herpesviruses have evolved a variety of mechanisms to modulate HDACs functions. It has been reported that varicella zoster virus (VZV) encoded ORF66 protein kinase (the U_S_3 ortholog in VZV) phosphorylates HDAC1 at serine 406 (S406) and HDAC2 at S407 which are unique to cellular protein kinase target sites^20^, however, the biological functions of this phosphorylation are still not clear. In addition, EBV encoded EBNA3C and KSHV encoded K-bZIP protein both have been reported to interact with HDAC1 and HDAC2 to regulate viral gene expression^30,36,37^. These observations prompted us to study the interaction between Meq and HDAC1 and 2.

To examine whether Meq associates with chHDAC1 and 2, we first visualized the subcellular localization of Meq and chHDAC1 and 2 in two MDV lymphoblastoid tumor cell lines. Co-localization of Meq and chHDAC1 and 2 was observed in the cell nuclei of MDV lymphoblastoid tumor cells (Fig. 1A). IP analysis further demonstrate that Meq physically associates with chHDAC1 and 2 in co-transfected 293T cells (Fig. 1B and Supplementary Fig. S2 online) and MDV lymphoblastoid tumor cells (Fig. 1C and Supplementary Fig. S2 online). In addition, we observed that Meq directly or indirectly interacts with other components of the chHDAC1 and 2 associated CoREST, NuRD, and Sin3 protein complexes (Fig. 1B, C, and Supplementary Fig. S2 online). We next identified the N-terminal homodimerization domain of chHDAC1 and 2 as essential for their interaction with Meq (Figs. 2 and 3, and Supplementary Fig. S3 and S4 online). Our results also showed that FLAG-chHDAC1 and FLAG-chHDAC2 could still efficiently co-precipitate HA tagged chHDAC1 and chHDAC2 in the presence of Meq (Fig. 3D, E, and Supplementary Fig. S4 online), indicating Meq does not interfere the homodimerization of chHDAC1 and chHDAC2. In addition, we showed that the BZIP domain of Meq is important for its interaction with chHDAC1 and 2 (Fig. 4 and Supplementary Fig. S5 online). Interestingly, we observed that N-120 of Meq strongly interacts with chHDAC2, but N-150 just slightly pulls down chHDAC2, suggesting that that amino acids 121–150 of Meq may interfere in its interaction with chHDAC2 (Fig. 4D and Supplementary Fig. S5 online). These results suggest that Meq may associate with chHDAC1 and chHDAC2 via different mechanisms despite the high homology between chHDAC1 and chHDAC2. Although, our study mapped the domains in Meq and chHDAC1 and 2 that are responsible for the interaction, we cannot rule out the possibility that the loss of interaction may due to the changes in their 3D conformation structure.

Another interesting point we noticed throughout our study is that overexpression of Meq resulted in lower levels of chHDAC1 and 2 (Fig. 3D, E, and Supplementary Fig. S4 online). This observation was confirmed by results showing that Meq induces the partial degradation of endogenous and exogenous chHDAC1 and 2 protein but does not affect the levels of mRNA expression (Fig. 5A–E, and Supplementary Fig. S6 online), and this process is specific to chHDAC1 and 2 as Meq did not degrade other interaction partners, including CREB and c-Jun (Fig. 5F and Supplementary Fig. S6 online). We then investigated the potential mechanisms utilized by Meq to facilitate the degradation of chHDAC1 and 2. The proteasome dependent pathway and autophagy are two major proteolytic pathways for protein degradation^38^. The proteasome is a protein complex that is present in both nucleus and cytoplasm, and which can eliminate misfolded or unnecessary proteins. The main proteasome is 26S which consists of a 20S core particle and two 19S regulatory particles^39^. Mostly, proteins targeted for degradation are dependent on ubiquitin conjugation, however there are exceptions of degradation via a ubiquitin independent proteasome degradation (UIPD) pathway^40^. Ubiquitination is a sequential procedure mediated by three enzymes: E1 ubiquitin activating enzyme, E2 ubiquitin conjugating enzymes, and E3 ubiquitin ligase enzyme. Autophagy is another proteolysis mechanism used by the cell to degrade unnecessary proteins, and consists of three different type: macroautophagy, microautophagy, and chaperone-mediated autophagy (CMA)^39^. Unlike ubiquitin dependent proteasome degradation, proteins eliminated by autophagy are indiscriminately degraded upon binding to lysosomes. A number of viral proteins have been showed to degrade cellular proteins via proteasome, and some viral proteins even contain E3 ubiquitin ligase domains. It has been shown that HSV-1 ICP0 has two distinct E3 ligase domains that play an important role in mediating ubiquitination and degradation^41^. KSHV encoded transcription activator (RTA) has been shown to act as an E3 ligase to ubiquitinate and degrades interferon regulatory factor 7 (IRF7) and KSHV-RTA binding protein (K-RBP)^42,43^. In addition, pp71, a transactivator encoded by HCMV, has been reported to degrade Rb and Daxx through UIPD^44^. With the treatment of proteasome inhibitor MG132, we showed that Meq mediates the partial degradation of chHDAC1 and 2 through a proteasome dependent pathway (Fig. 6 and Supplementary Fig. S7, S8 and S9 online). Considering the importance of chHDACs in gene expression regulation and MDV genome replication, we speculate that Meq is involved in transcriptional regulation and MDV replication through, at least partially, its role in manipulating chHDAC1 and 2. To further study the interplay between Meq and the cellular proteasome pathway, we found that ectopic expression of Meq reduces levels of global ubiquitinated proteins via the proteasome dependent pathway, for which amino acids 51–120 of Meq are important (Fig. 7 and Supplementary Fig. S10 online). This reduction could be due to Meq mediated degradation of ubiquitinated proteins or to the reduction of ubiquitination process in general. In this study, Meq from a very virulent plus MDV, strain 686, was used. As Meq is highly conserved between different strains of MDV, it is highly possible that Meq from other pathotypes of MDV shares the ability to interact with and degrade chHDAC1 and 2. The proteasome dependent degradation pathway is a complex sequential process, in which a large number of cellular components are involved. Further studies will be needed to reveal exact mechanisms behind the interplay between MDV Meq and the proteasome dependent degradation pathway and its role in MDV pathogenesis.

## Materials and methods

### Cells

Human embryonic kidney 293T cells and chicken DF-1 cells were grown in Dulbecco’s modified Eagle medium (DMEM) supplemented with 10% fetal bovine serum (FBS). MDV lymphoblastoid tumor cell lines, MSB-1^45^ and MKT-1 (MDV derived kidney T cell line)^46^, were grown in RPMI 1640 medium supplemented with 15% FBS. MKT-1 cell line was established from a kidney lymphoma of chickens infected with a very virulent strain of MDV^46^. All cells were grown at 37 °C in the presence of 5% CO_2._ 293T and DF-1 cells were used for transient transfections, and MDV tumor cell lines were used for immunofluorescence and immunoprecipitation assays.

### Chemicals

Cycloheximide (CHX) and MG132 were purchased from Millipore-Sigma and reconstituted in dimethyl sulfoxide (DMSO) to prepare stock solution according to manufacturer’s instruction. CHX was used to determine the protein half-life and MG132 was used to study the proteasome degradation pathway.

### Plasmids construction

pcDNA3.1/Zeo ( +) mammalian expression vector (Invitrogen) was used for the generation of Meq and chHDAC1 and 2 constructs. All primers are listed in Table S1 and all cloned genes were validated by sequencing.

#### Meq plasmids

FLAG, HA, and T7 tagged full length Meq was amplified from MDV (strain 686) bacterial artificial chromosome (BAC) DNA^47^ using primers 1 to 4 (Supplementary Table S1 online). The PCR products were purified using QIAEX II Gel Extraction Kit (Qiagen) followed by digestion and cloning into pcDNA3.1/Zeo (+) plasmid. Same processes were performed to generate FLAG tagged N- and C-terminal truncate plasmids using primers 1 and 5 to 16 (Supplementary Table S1 online). Overlapping PCR^48^ was performed to generate FLAG tagged BR, ZIP, and BZIP deletion Meq expression plasmids using primers 1, 4, and 17 to 22 (Supplementary Table S1 online).

#### chHDAC1 plasmids

FLAG and HA tagged full length chHDAC1 was amplified from chicken cDNA using primers 23 to 25 (Supplementary Table S1 online), followed by digestion and cloning into pcDNA3.1/Zeo (+) plasmid. Same experiments were performed using primers 23 and 26 to 33 (Supplementary Table S1 online) to generate FLAG tagged N- and C-terminal truncated chHDAC1 constructs.

#### chHDAC2 plasmids

FLAG and HA tagged full length chHDAC2 was amplified from chicken cDNA using primers 34 to 36 (Supplementary Table S1 online), followed by digestion and cloning into pcDNA3.1/Zeo (+) plasmid. FLAG tagged N- and C-terminal truncated chHDAC2 constructs were generated using primers 34 and 37 to 42 (Supplementary Table S1 online).

### Immunofluorescence assay (IFA)

To examine the co-localization of Meq with chHDAC1 and 2, IFA was performed using MDV lymphoblastoid tumor cell lines. MSB-1 and MKT-1 tumor cells were pelleted by centrifuge at 500×*g* for 5 min followed by three washes with phosphate buffered saline (PBS). Cell pellets were resuspended with 200 μl PBS and settled onto coverslips for 2 min at room temperature. Then, cells were fixed with 3.7% formaldehyde-PBS solution followed by quenching with 100 mM glycine-PBS solution for 5 min and permeabilized with acetone-methanol (1:1) solution for 15 min at room temperature. After three washes with PBS, cells were incubated with mouse anti-HDAC1 (Santa cruz biotechnology) or mouse anti-HDAC2 (Santa cruz biotechnology) and rabbit anti-Meq (kindly provided by Dr. Hsing-Jien Kung) antibodies for 1 h, followed by another hour incubation with goat anti-mouse-Texas Red antibody and goat anti-rabbit-Alex Flour 488 antibody at room temperature. After three washes with PBS, cells were stained with 4′,6-diamidino-2-phenylindole (DAPI) for 5 min at room temperature. Cells on coverslips were then mounted on glass slides with ProLong Diamond Antifade Mountant (Thermo Fisher Scientific) and imaged with Zeiss LSM 780 NLO Multiphoton Microscope.

### Immunoprecipitation (IP) and Western blot (WB)

To study the interactions between Meq and chHDAC1 and 2, IP and WB were performed with MDV lymphoblastoid tumor cell lines and transfected 293T cells.

#### MDV lymphoblastoid tumor cells

MDV lymphoblastoid tumor cells were lysed in EBC lysis buffer (50 mM Tris–HCl, 120 mM NaCl, 0.5% NP-40, 50 mM NaF, 200 μM Na2VO4) with 1 mM phenylmethylsulfonyl fluoride (PMSF) and additional protease inhibitor cocktail (Thermo Fisher Scientific). For IP, 500 µg cell lysates were gently rotated overnight in the presence of 2 µg rabbit anti-Meq antibody or normal rabbit IgG (Cell Signaling Technology) at 4 °C. Next day, the immune complexes were incubated with protein A and protein G Sepharose beads (Invitrogen) mixture for 2 h at 4 °C. After five washes with EBC lysis buffer, proteins were eluted in 2 × sodium dodecyl sulfate (SDS) buffer and boiled for 5 min. The immunoprecipitated samples and 10% input (50 µg cell lysates) were subjected to SDS–polyacrylamide gel electrophoresis (PAGE) and then transferred to polyvinylidene fluoride (PVDF) membrane (Millipore-Sigma). PVDF membranes were incubated with 5% nonfat milk at room temperature for 1 h, followed by WB with primary antibody incubation overnight at 4 °C and horseradish peroxidase (HRP) conjugated secondary antibody incubation 1 h at room temperature. After three washes with PBST (0.1% Tween 20), PVDF membranes were visualized with Super Signal West Pico PLUS Chemiluminescent Substrate (Thermo Fisher Scientific).

#### 293T cells

The indicated plasmids (4–6 mg per 2 million cells) were transiently transfected into 293T or DF-1 cells using polyethylenimine (PEI) (1 mg/ml). Forty-eight hours later, transfected 293T or DF-1 cells were lysed in EBC lysis buffer^49^ for general IP or SDS lysis buffer (1% SDS, 50 mM Tris HCl, 10% glycerol) for detection of ubiquitinated proteins. 500 µg cell lysates were incubated overnight with mouse anti-FLAG agarose beads (Sigma) at 4 °C with gentle rotation. The subsequent IP and WB processes were carried out as described above.

Quantification of WB bands intensity was performed with Image J software.

### Dual luciferase assay

To determine the effect of Meq and chHDAC1 & 2 interactions in the transcriptional regulation of viral promoters, dual luciferase assay was performed with pGL3 luciferase vector (Promega) containing MDV *pp14* and *pp38* promoter as described previously^13^. Briefly, 293T cells were transfected with pcDNA expression plasmids and pGL3-*pp14*_promoter or pGL3-*pp38*_promoter and *renilla* luciferase vector. After 48 h, cells were lysed with passive lysis buffer, followed by *Firefly* and *renilla* luciferase activity measurement. Experiments were repeated three times in triplicate. Results were presented as average fold change relative to values derived from pcDNA empty vector (Ev) transfected cells, with error bars representing standard deviation (SD). The statistical differences were analyzed by Student *t* test. NS: not significant, *: p < 0.05, **: p < 0.01.

### RNA isolation and quantitative reverse transcriptase polymerase chain reaction (qRT-PCR)

To determine the effect of Meq in the transcription of chHDAC1 and 2, DF-1 cells were transfected with pcDNA-FLAG-Meq or pcDNA empty vector (Ev). Forty-eight hours later, cells were harvested for RNA isolation using PureLink RNA Mini Kit (Invitrogen) as per manufacturer's instructions, followed by cDNA systhesis. qRT-PCR reactions, including melt cure analysis, were processed on a CFX96 Real time PCR Detection System (Bio-Rad) using iTaq Universal SYBR Green Supermix (Bio-Rad) with primers 43 to 48 (Table S1). Gene expression was normalized to signal of chicken *GAPDH*, and qRT-PCR results were analyzed using the 2^-ΔΔCT^ method. Fold changes were calculated as to values derived from Ev transfected cells, and presented as average of three independent cell culture experiments with error bars representing standard deviation (SD). The statistical differences were analyzed by Student *t* test.

All methods were carried out in accordance with relevant guidelines and regulations of Texas A&M University Institutional Biosafety Committee (IBC).

## Supplementary Information

Supplementary Information.
