# Supplementary material for: Marek’s disease virus Meq oncoprotein interacts with chicken HDAC 1 and 2 and mediates their degradation via proteasome dependent pathway
Source: Sci Rep. 2021 Jan 12;11:637. doi: 10.1038/s41598-020-80792-2 (PMC7803728; doi:10.1038/s41598-020-80792-2)
Supplement: Supplementary file 1 — Supplementary Information. [file 41598_2020_80792_MOESM1_ESM.pdf]

Marek's disease virus Meq oncoprotein interacts with chicken HDAC 1 and 2 and mediates their degradation via proteasome dependent pathway

Yifei Liao[1], Blanca Lupiani[1], Yoshihiro Izumiya[2] and Sanjay M. Reddy[1, *]

1. Department of Veterinary Pathobiology, College of Veterinary Medicine & Biomedical Sciences, Texas A&M University, College Station, Texas, USA.
2. Department of Dermatology, School of Medicine, University of California, Davis, Sacramento, California, USA.

* Corresponding author

Sanjay M. Reddy

Department of Veterinary Pathobiology

College of Veterinary Medicine & Biomedical Sciences

MS4467, TAMU

College Station, Texas, USA, 77843

Phone: 979-458-0658

E-mail: SReddy@cvm.tamu.edu

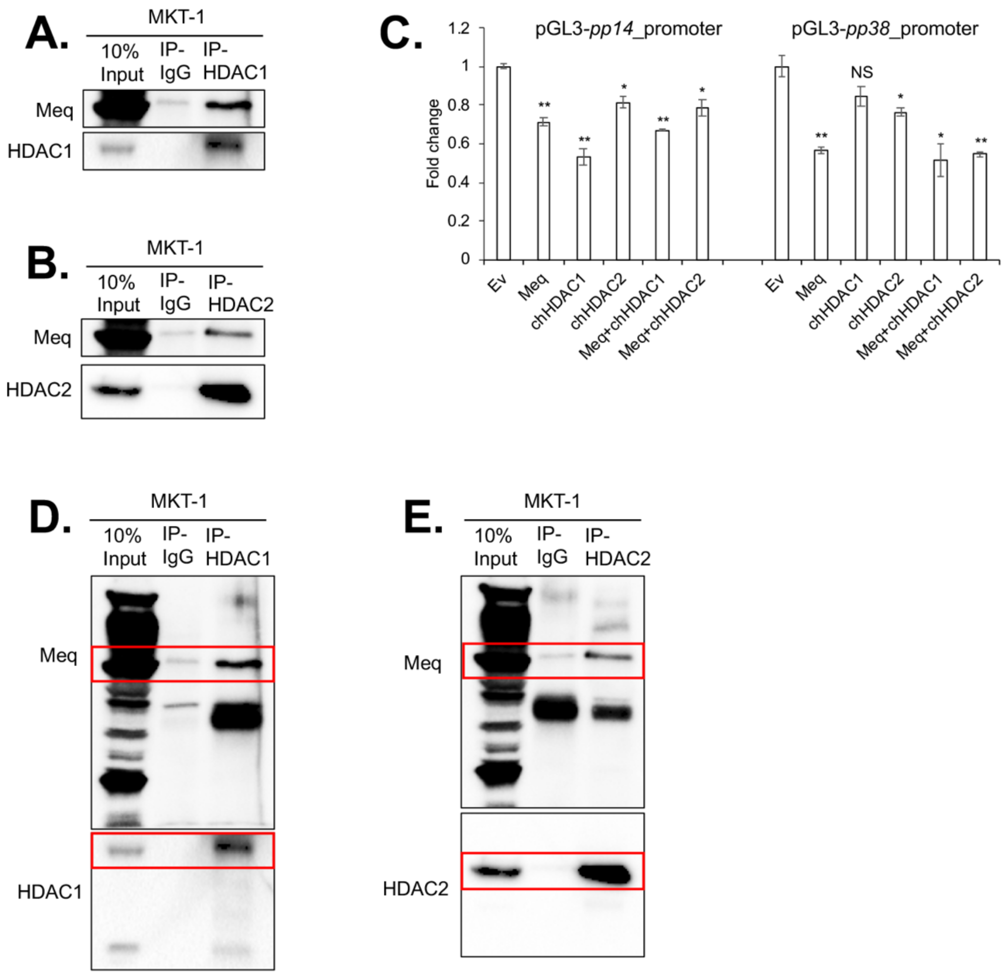

Figure S1. chHDAC1 and 2 interacts with MDV Meq. MKT-1 tumor cells were lysed and subjected to immunoprecipitation with rabbit anti-HDAC1 (A) or rabbit anti-HDAC2 (B) and normal rabbit IgG, followed by Western blot analysis with the indicated antibodies. (C) pcDNA expression plasmids were co-transfected with pGL3-*pp14*_promoter (left) or pGL3-*pp38*_promoter (right) and renilla luciferase vector into 293T cells. 48 hours after transfection, Firefly and renillla luciferase activity were measured using Dual-Glo® Luciferase Assay System as per manufacturer's protocol. The experiment was repeated three times in triplicate. Error bars indicate standard deviation (SD). The statistical differences were analyzed by Student *t* test. NS: not significant, *: $p<0.05$, **: $p<0.01$. (D) Original blots related to Fig. S1A. (E) Original blots related to Fig. S1B.

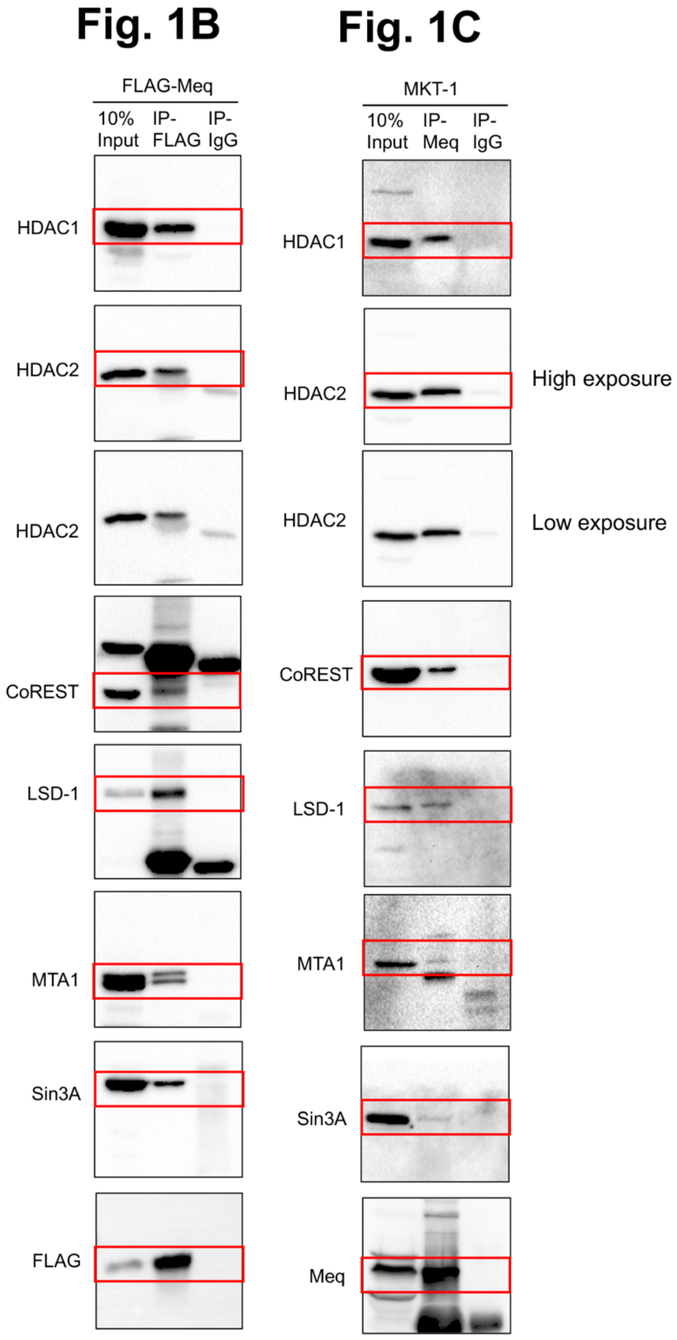

Figure S2. MDV Meq interacts with chHDAC1 and 2. Original blots related to Fig. 1B and 1C. (Fig. 1B) 293T cells were transfected with pcDNA-FLAG-Meq. Forty-eight hours later, whole cell lysates were subjected to immunoprecipitation (IP) with FLAG antibody and normal mouse IgG. Western blot (WB) analysis was performed with the indicated antibodies targetting HDAC1, HDAC2 and other components of the CoREST, NuRD, and Sin3 protein complexes. (Fig. 1C) MKT-1 tumor cells were lysed and subjected to IP with Meq polyclonal antibody and normal rabbit IgG, followed by WB with the indicated antibodies.

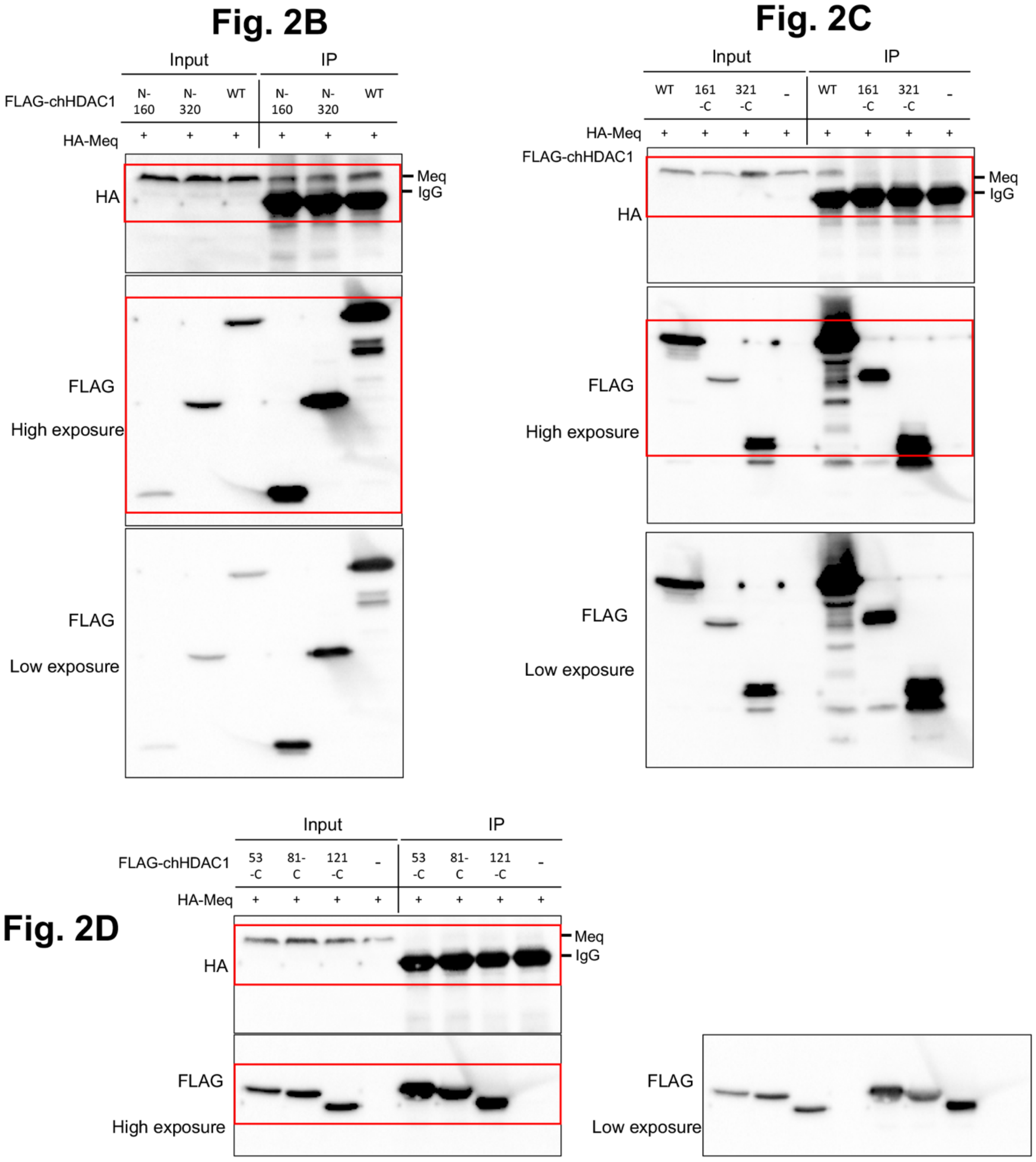

Figure S3. Mapping the domain of chHDAC1 that interacts with MDV Meq. Related to Fig. 2. Original blots related to Fig. 2B, 2C, and 2D. (Fig. 2B, 2C, 2D). pcDNA-FLAG-chHDAC1 deletion mutants were co-transfected with pcDNA-HA-Meq into 293T cells. Cells were lysed 48 hours post transfection and subjected to immunoprecipitation with mouse anti-FLAG agarose beads. Western blot was processed with HA and FLAG antibodies.

Fig. 3B

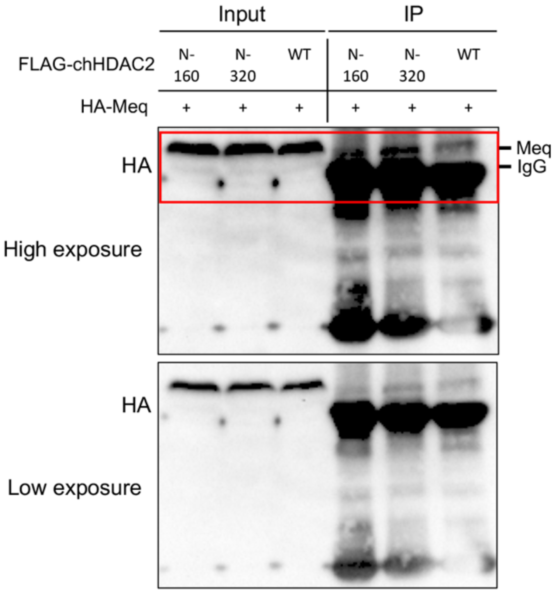

Fig. 3D

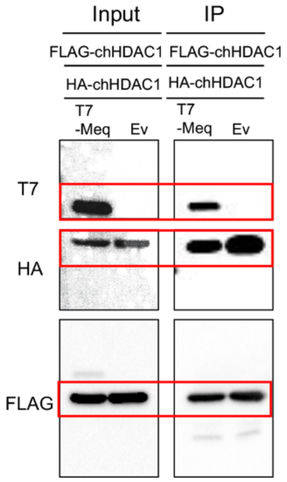

Fig. 3E

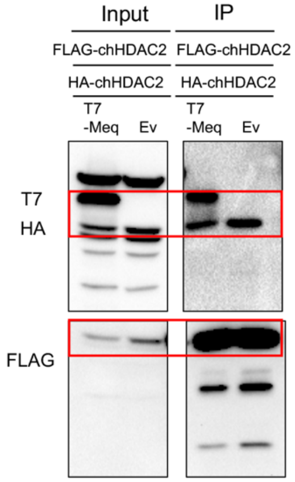

Fig. 3C

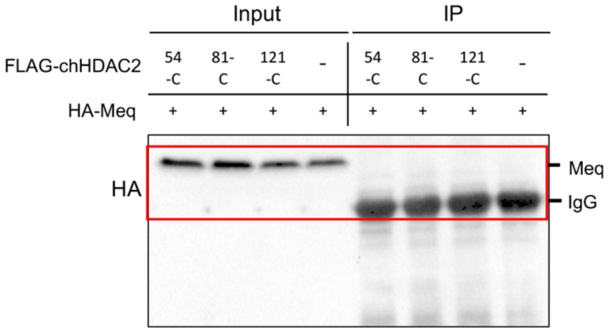

Figure S4. Mapping the domain of chHDAC2 that interacts with MDV Meq. Original blots related to Fig. 3B, 3C, 3D, and 3E. (Fig. 3B, 3C) pcDNA-FLAG-chHDAC2 deletion mutants were co-transfected with pcDNA-HA-Meq into 293T cells for 48 hours. Immunoprecipitation (IP) was performed with mouse anti-FLAG agarose beads, followed by Western blot (WB) analysis with HA antibody. pcDNA-T7-Meq or pcDNA empty vector (Ev) were co-transfected with pcDNA-FLAG-chHDAC1 and pcDNA-HA-chHDAC1 (Fig. 3D) or pcDNA-FLAG-chHDAC2 and pcDNA-HA-chHDAC2 (Fig. 3E) into 293T cells. Forty-eight hours later, IP was processed with FLAG antibody and normal mouse IgG, followed by WB with T7, HA, and FLAG antibodies.

Fig. 4B

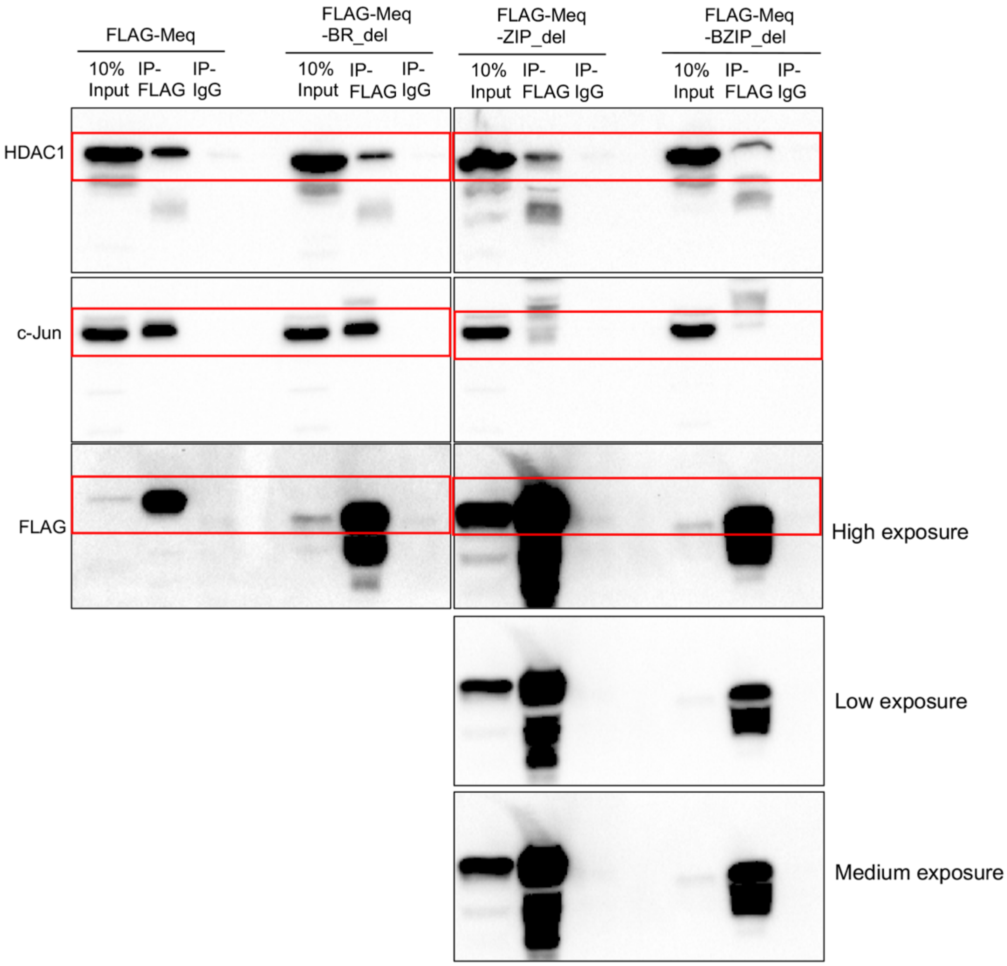

FLAG-Meq
FLAG-Meq -BR_del
FLAG-Meq -ZIP_del
FLAG-Meq -BZIP_del
10% Input
IP-FLAG
IP-IgG
HDAC1
c-Jun
FLAG
High exposure
Low exposure
Medium exposure

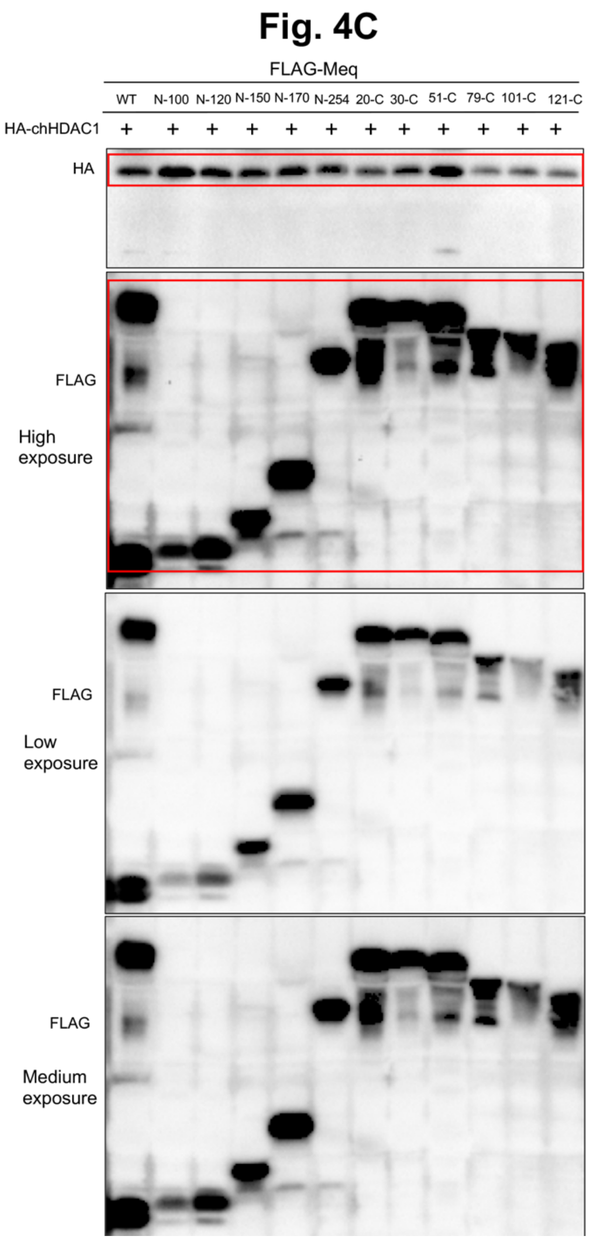

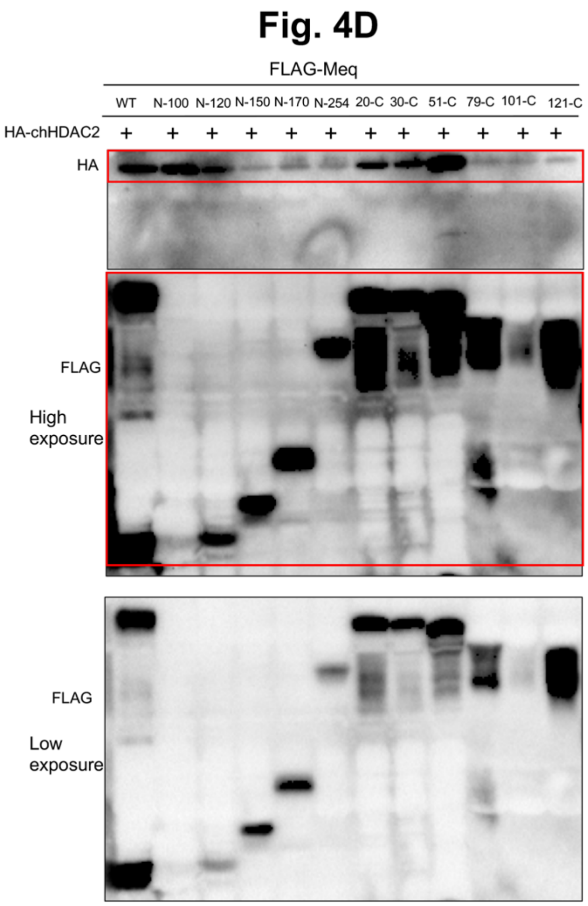

Figure S5. Mapping the domain of Meq that interacts with chHDAC1 and chHDAC2. Original blots related to Fig. 4B, 4C, and 4D. (Fig. 4B) pcDNA-FLAG-Meq deletion mutants were transfected into 293T cells for 48 hours. Whole cell lysates were subjected to immunoprecipitation (IP) with rabbit anti-FLAG antibody or normal rabbit IgG, followed by Western blot (WB) analysis with HDAC1, c-Jun, and FLAG antibodies. pcDNA-Meq deletion mutants were co-transfected with pcDNA-HA-chHDAC1 (Fig. 4C) or pcDNA-HA-chHDAC2 (Fig. 4D) into 293T cells. Whole cell lysates were harvested 48 hours post transfection and subjected to IP with mouse anti-FLAG agarose beads. WB was processed with HA and FLAG antibodies.

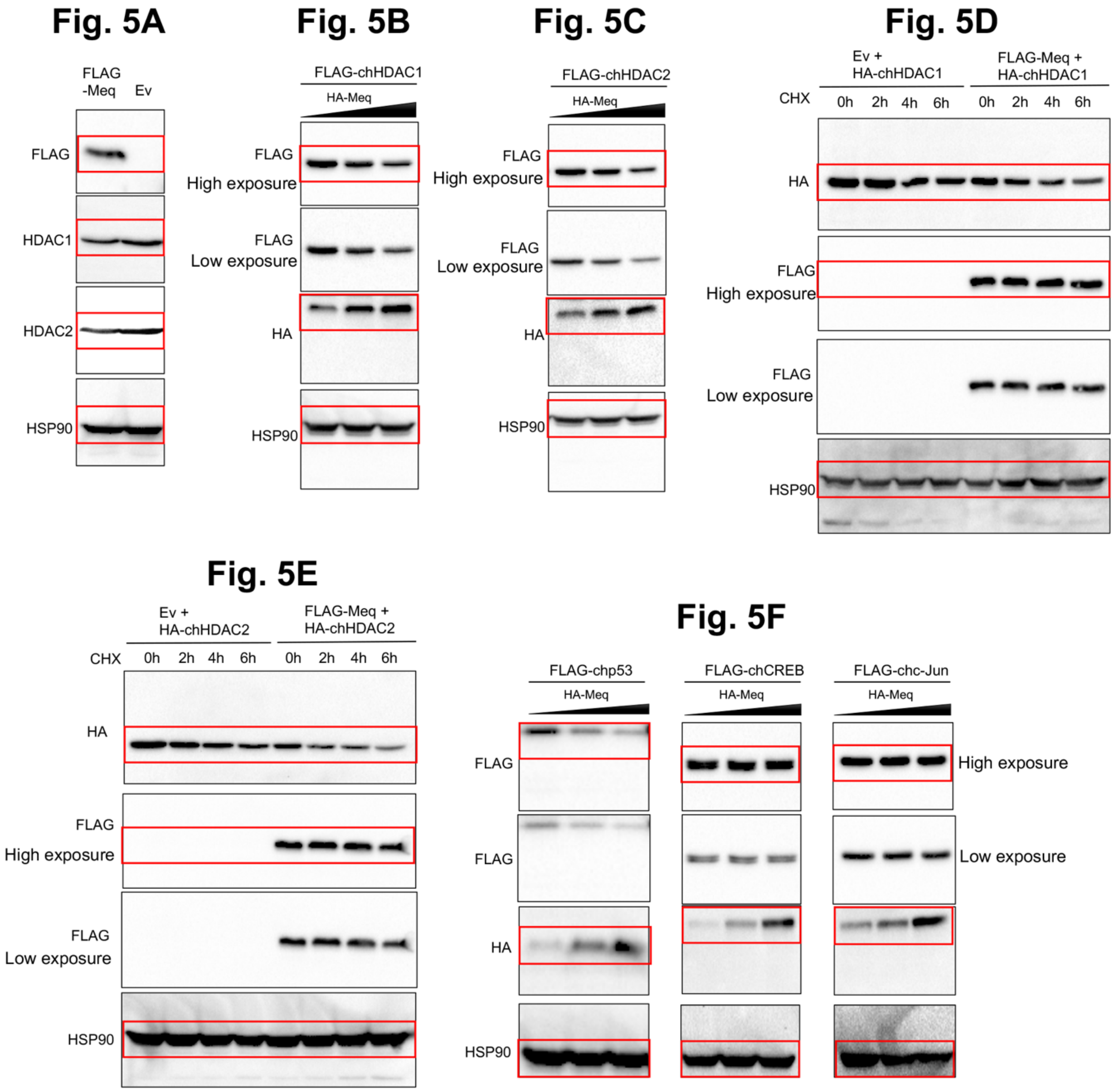

Fig. 5A
FLAG -Meq
Ev
FLAG
HDAC1
HDAC2
HSP90
Fig. 5B
FLAG-chHDAC1
HA-Meq
FLAG High exposure
FLAG Low exposure
HA
HSP90
Fig. 5C
FLAG-chHDAC2
HA-Meq
FLAG High exposure
FLAG Low exposure
HA
HSP90
Fig. 5D
Ev + HA-chHDAC1
FLAG-Meq + HA-chHDAC1
CHX
0h 2h 4h 6h 0h 2h 4h 6h
HA
FLAG High exposure
FLAG Low exposure
HSP90
Fig. 5E
Ev + HA-chHDAC2
FLAG-Meq + HA-chHDAC2
CHX
0h 2h 4h 6h 0h 2h 4h 6h
HA
FLAG High exposure
FLAG Low exposure
HSP90
Fig. 5F
FLAG-chp53
FLAG-chCREB
FLAG-chc-Jun
HA-Meq
FLAG
High exposure
FLAG
Low exposure
HA
HSP90

Figure S6. MDV Meq mediates the degradation of chHDAC1 and 2. Original blots related to Fig. 5. (Fig. 5A) DF-1 cells were transfected with pcDNA-FLAG-Meq or pcDNA empty vector (Ev) and 48 hours later, cells were harvested for protein and RNA extraction. Western blot (WB) analysis was processed with the indicated antibodies (left) and quantified with Image J and presented as fold change compared to Ev (middle). qRT-PCR was processed with primers targeting chHDAC1 and chHDAC2 and presented as fold change compared to Ev (right). pcDNA-FLAG-chHDAC1 (Fig. 5B) or pcDNA-FLAG-chHDAC2 (Fig. 5C) were co-transfected with different amounts of pcDNA-HA-Meq into 293T cells for 48 hours. Whole cell lysates were subjected to WB with the indicated antibodies (left). WB results were quantified with Image J, normalized to HSP90, and presented as fold change compared to the least amount of Meq transfection (right). pcDNA-FLAG-Meq or pcDNA Ev were cotransfected with pcDNA-HA-chHDAC1 (Fig. 5D) or pcDNA-HA-chHDAC2 (Fig. 5E) into 293T cells and 24 hours later, cells were treated with cycloheximide (CHX, 1 mg/ml) for the indicated length of time. WB were performed with HA, FLAG, and HSP90 antibodies (upper). HA-chHDAC1 or HA-chHDAC2 protein levels were quantified with Image J, normalized to HSP90, and presented as fold change compared to non-treated cells (bottom). All experiments were repeated two times. Error bars indicate standard deviation (SD). (Fig. 5F) pcDNA-FLAG-chp53, pcDNA-FLAG-chCREB, or pcDNA-FLAG-chc-Jun were co-transfected with different amounts of pcDNA-HA-Meq into 293T cells and 48 hours later, whole cell lysates were subjected to WB with the indicated antibodies. The statistical differences were analyzed by Student *t* test. *: $p<0.05$, **: $p<0.01$, ***: $p<0.001$.

Fig. 6A

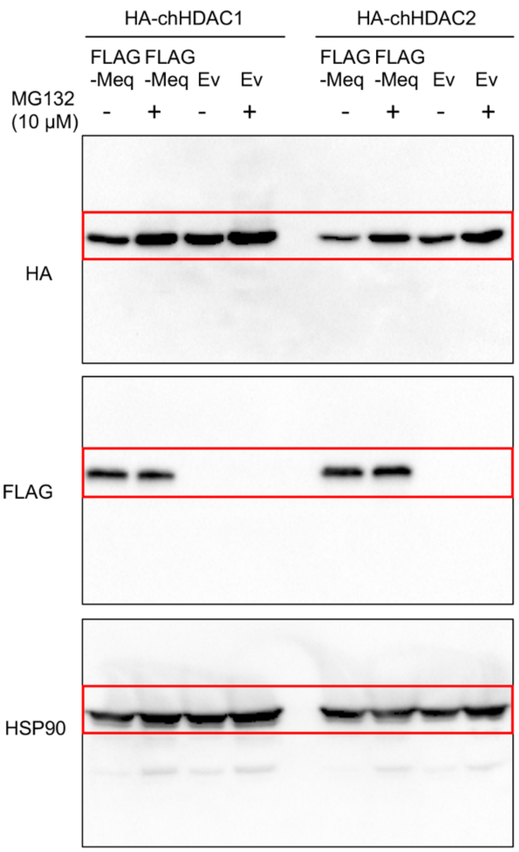

Figure S7. MDV Meq mediates the partial degradation of chHDAC1 and 2 via the proteasome dependent pathway. Original blots related to Fig. 6A. (Fig. 6A) pcDNA-HA-chHDAC1 or pcDNA-HA-chHDAC2 were co-transfected with pcDNA-FLAG-Meq or pcDNA empty vector (Ev) into 293T cells and 24 hours later, cells were treated overnight with or without MG132 (10 μM). Western blot (WB) analysis was performed with whole cell lysates using the indicated antibodies. Representative WB images are shown (upper). Protein levels of HA-chHDAC1 or HA-chHDAC2 were quantified with Image J, normalized to HSP90, and presented as fold change compared to MG132 treated pcDNA-HA-chHDAC1 or pcDNA-HA-chHDAC2 and pcDNA Ev co-transfected cells (bottom). The statistical differences were analyzed by Student *t* test. *: $p<0.05$, NS: not significant.

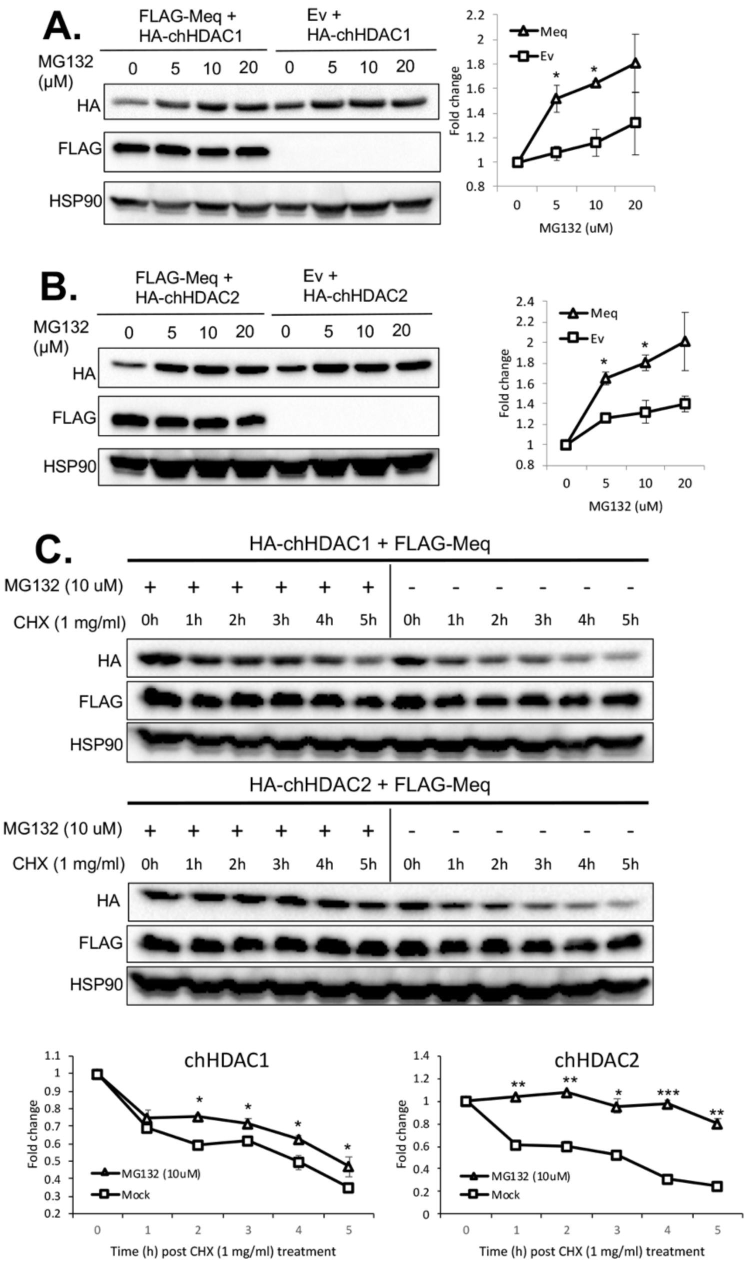
A.
FLAG-Meq + HA-chHDAC1
Ev + HA-chHDAC1
MG132 (μM)
0 5 10 20 0 5 10 20
HA
FLAG
HSP90
Meq
Ev
Fold change
MG132 (uM)
B.
FLAG-Meq + HA-chHDAC2
Ev + HA-chHDAC2
MG132 (μM)
0 5 10 20 0 5 10 20
HA
FLAG
HSP90
Meq
Ev
Fold change
MG132 (uM)
C.
HA-chHDAC1 + FLAG-Meq
MG132 (10 uM)
CHX (1 mg/ml)
0h 1h 2h 3h 4h 5h 0h 1h 2h 3h 4h 5h
HA
FLAG
HSP90
HA-chHDAC2 + FLAG-Meq
MG132 (10 uM)
CHX (1 mg/ml)
0h 1h 2h 3h 4h 5h 0h 1h 2h 3h 4h 5h
HA
FLAG
HSP90
chHDAC1
MG132 (10uM)
Mock
Fold change
Time (h) post CHX (1 mg/ml) treatment
chHDAC2
MG132 (10uM)
Mock
Fold change
Time (h) post CHX (1 mg/ml) treatment

Figure S8. MG132 treatment inhibits the degradation of chHDAC1 and 2 induced by Meq. pcDNA-FLAG-Meq or pcDNA empty vector (Ev) were cotransfected with pcDNA-HA-chHDAC1 (A) or pcDNA-HA-chHDAC2 (B) into 293T cells and 24 hours post transfection, cells were treated with different concentrations of MG132 overnight. Western blot (WB) analysis was performed with HA, FLAG, and HSP90 antibodies (left) and quantified by Image J. Levels of HA-chHDAC1 or HA-chHDAC2 were normalized to HPS90 and presented as fold change compared to non-treated cells (right). (C) pcDNA-FLAG-Meq was co-transfected with pcDNA-HA-chHDAC1 or pcDNA-HA-chHDAC2 into 293T cells and 24 hours post transfection, cells were treated with MG132 (10 μM) overnight. Next day, cells were treated with cycloheximide (CHX, 1 mg/ml) for the indicated length of time, in the presence of MG132 (10 μM). WB analysis was performed with HA, FLAG, and HSP90 antibodies. Levels of HA-chHDAC1 or HA-chHDAC2 were quantified and normalized to HPS90, and presented as fold change compared to no CHX treated cells. Error bars indicate standard deviation (SD). All experiments were repeated at least two times. The statistical differences were analyzed by Student *t* test. *: $p<0.05$, *: $p<0.01$, *: $p<0.001$.

Fig. S8A

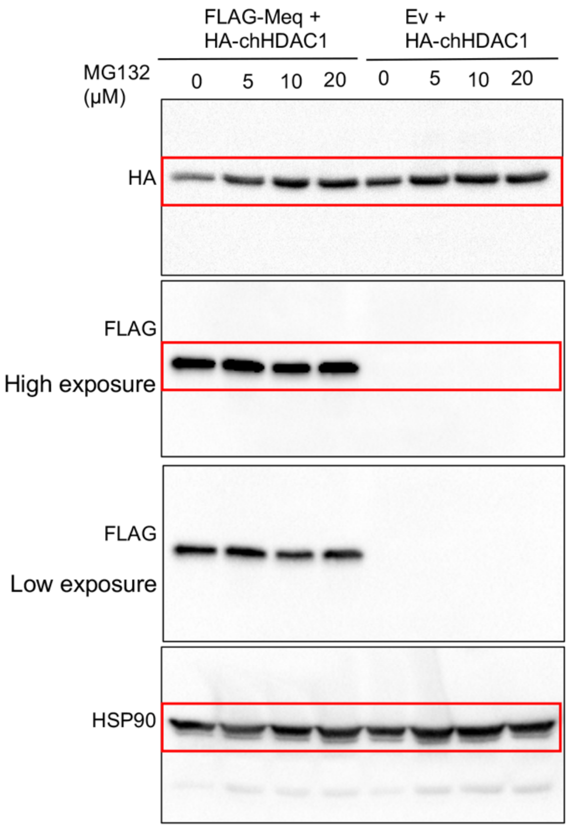

Fig. S8B

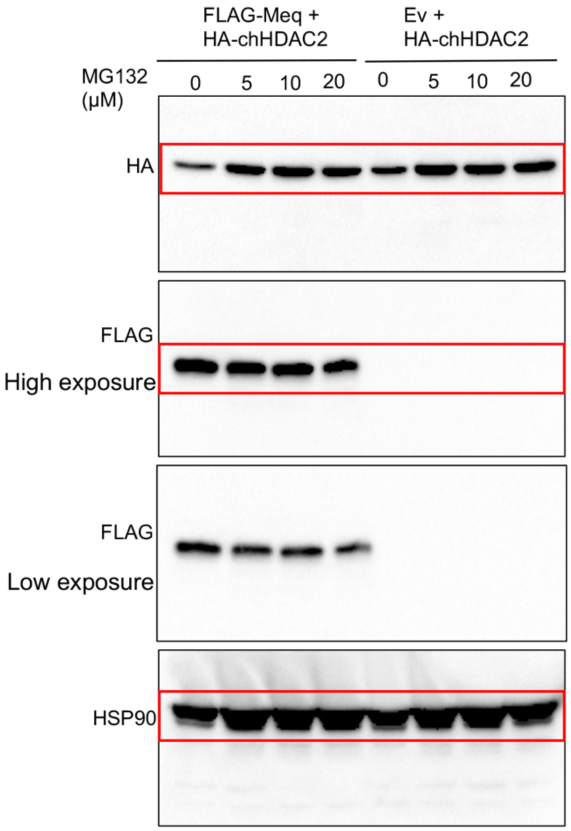

Fig. S8C

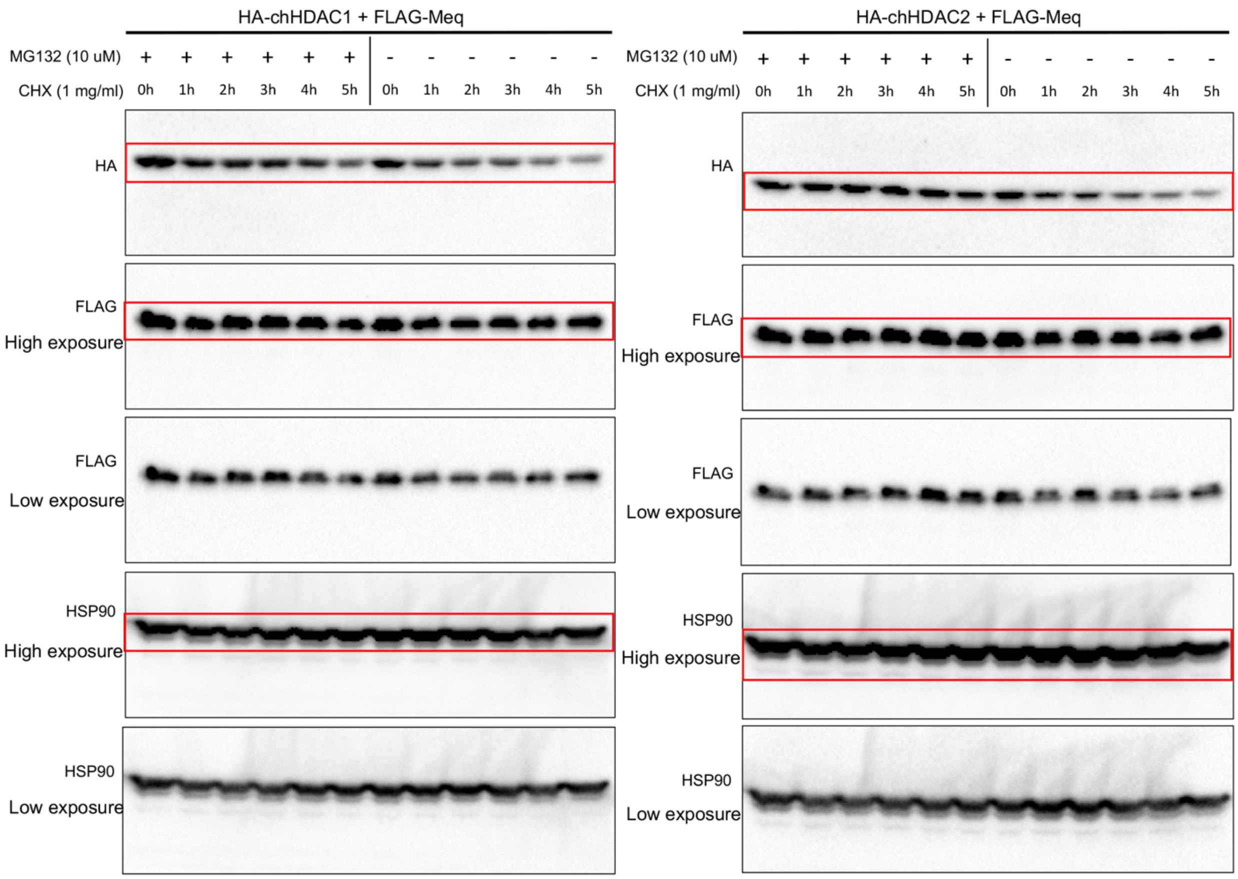

Figure S9. MG132 treatment inhibits the degradation of chHDAC1 and 2 induced by Meq. Original blots related to Fig. S8. pcDNA-FLAG-Meq or pcDNA empty vector (Ev) were cotransfected with pcDNA-HA-chHDAC1 (Fig. S8A) or pcDNA-HA-chHDAC2 (Fig. S8B) into 293T cells and 24 hours post transfection, cells were treated with different concentrations of MG132 overnight. Western blot (WB) analysis was performed with HA, FLAG, and HSP90 antibodies. (Fig. S8C) pcDNA- FLAG-Meq was co-transfected with pcDNA-HA-chHDAC1 or pcDNA-HA-chHDAC2 into 293T cells and 24 hours post transfection, cells were treated with MG132 (10 μM) overnight. Next day, cells were treated with cycloheximide (CHX, 1 mg/ml) for the indicated length of time, in the presence of MG132 (10 μM). WB analysis was performed with HA, FLAG, and HSP90 antibodies.

Fig. 7A

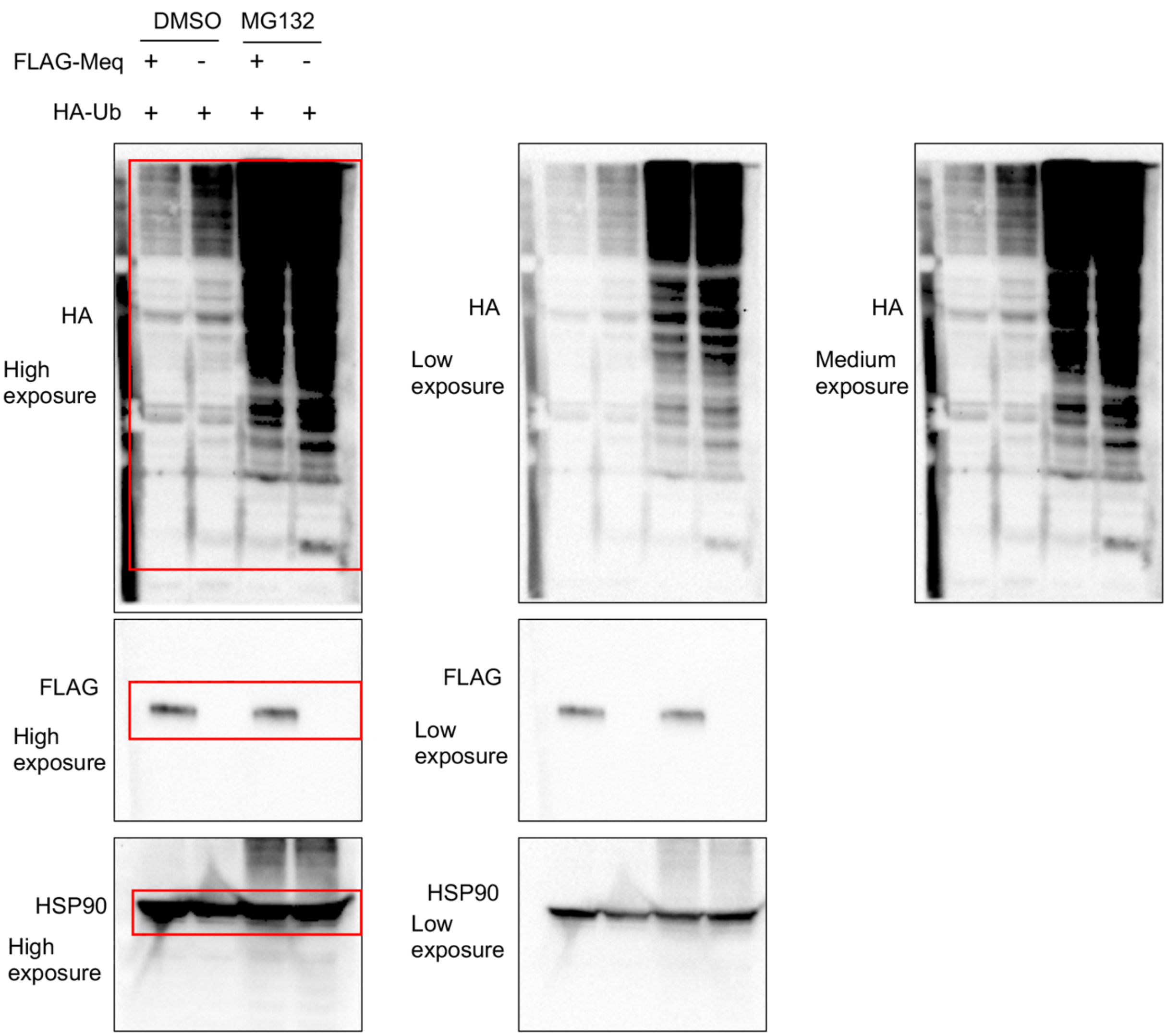
DMSO
MG132
FLAG-Meq + - + -
HA-Ub + + + +
HA
High exposure
HA
Low exposure
HA
Medium exposure
FLAG
High exposure
FLAG
Low exposure
HSP90
High exposure
HSP90
Low exposure

Fig. 7B

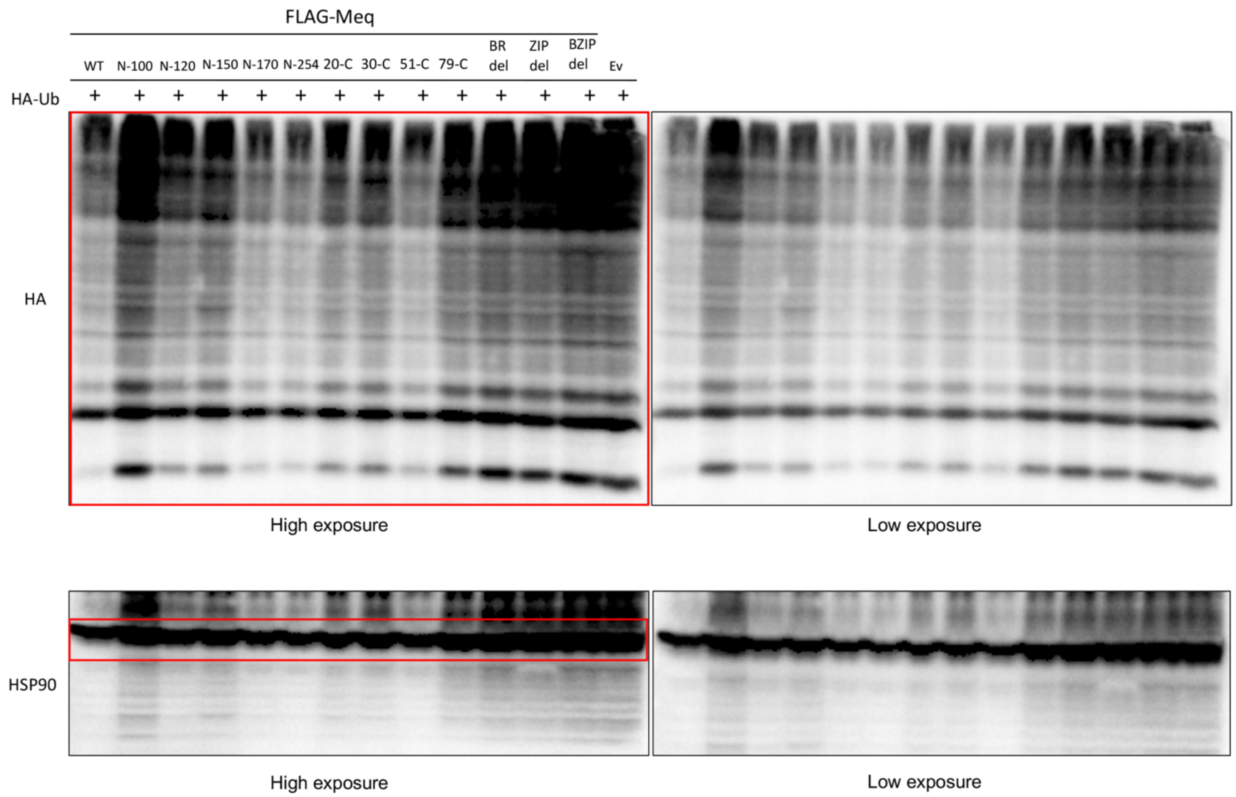
FLAG-Meq
WT N-100 N-120 N-150 N-170 N-254 20-C 30-C 51-C 79-C BR del ZIP del BZIP del Ev
HA-Ub + + + + + + + + + + + + + +
HA
High exposure
Low exposure
HSP90
High exposure
Low exposure

Fig. 7C

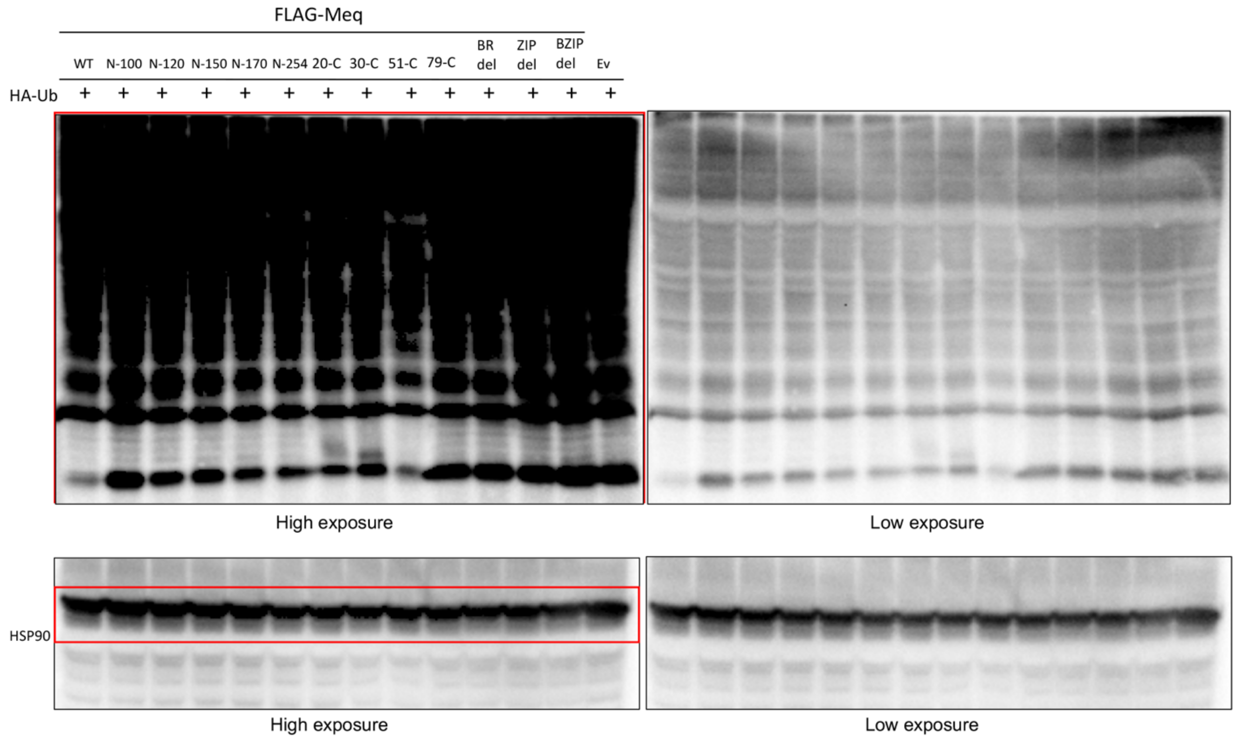
FLAG-Meq
WT N-100 N-120 N-150 N-170 N-254 20-C 30-C 51-C 79-C BR del ZIP del BZIP del Ev
HA-Ub + + + + + + + + + + + + + +
High exposure
Low exposure
HSP90
High exposure
Low exposure

Figure S10. MDV Meq mediates the degradation of global ubiquitinated proteins via the proteasome dependent pathway. Original blots related to Fig. 7. (Fig. 7A) pcDNA-FLAG-Meq or pcDNA empty vector (Ev) were co-transfected with pcDNA-HA-Ub into 293T cells. Twenty-four hours later, cells were treated with DMSO or MG132 (10 μM) overnight. Cells were lysed and subjected to Western blot (WB) analysis with HA, FLAG, and HSP90 antibodies. (Fig. 7B) pcDNA-HA-Ub was cotransfected with the indicated pcDNA-FLAG-Meq deletion mutants or pcDNA Ev into 293T cells and 48 hours later, cells were lysed and subjected to WB with HA and HSP90 antibodies. (Fig. 7C) pcDNA-HA-Ub was co-transfected with the indicated pcDNA-FLAG-Meq deletion mutants or pcDNA Ev to 293T cells. Twenty-four hours later, cells were treated with MG132 (10 μM) overnight. Cells were lysed and subjected to WB with HA and HSP90 antibodies.

Table S1. List of primers used in pcDNA plasmid constructions.

**Primer number**	**Primer name**	**Sequence (5’ to 3’)**
1	Meq-FLAG-F	TC**GCTAGC**ATG***GACTACAAAGACGATGACGACAAG***TCTCAGGAGCCAGAGCCG
2	Meq-HA-F	TC**GCTAGC**ATG***TACCCATACGATGTTCCAGATTACGCT***TCTCAGGAGCCAGAGCCG
3	Meq-T7-F	TC**GCTAGC**ATG***ATGGCTAGCATGACTGGTGGACAGCAAATGGGT***TCTCAGGAGCCAGAGCCG
4	Meq-R	GATC**GCGGCCGC**TCAGGGTCTCCCGTCACCTG
5	Meq-N-100-R	gatc**GCGGCCGC**tcaACGTAGGTGTTCATTGGCCCT
6	Meq-N-120-R	gatc**GCGGCCGC**tcaATGACGAGCCAACTGTACACG
7	Meq-N-150-R	gatc**GCGGCCGC**tcaGCAAATGGGAGGTTCAGGAAC
8	Meq-N-170-R	gatc**GCGGCCGC**tcaAGGTTGGGAACCGGAGCAATG
9	Meq-N-254-R	gatc**GCGGCCGC**tcaGATGCCCTCCGGAGATGGAGG
10	Meq-21-C-F	gatc**GCTAGC**atgCCCCTCGATCTTTCTCTCGGG
11	Meq-31-C-F	gatc**GCTAGC**atgAGACGGAAAAAAAGGAAAAGT
12	Meq-51-C-F	gatc**GCTAGC**atgGACGGCCTATCTGAGGAGGAG
13	Meq-79-C-F	gatc**GCTAGC**atgCAGACGGACTATGTAGACAAA
14	Meq-101-C-F	gatc**GCTAGC**atgAAGGAAATTCGAGATCTAAGG
15	Meq-121-C-F	gatc**GCTAGC**atgGAGCCAGTTTGCCCTATGGCG
16	Meq-FLAG-R	TC**GCGGCCGC**TCA***CTTGTCGTCATCGTCTTTGTAGTC*** GGGTCTCCCGTCACCTG
17	Meq-BR_del-F	TTTCTCTCGGGTCGACTTCGCTCCATGAAGCATGTGAAGA
18	Meq-BR_del-R	TCTTCACATGCTTCATGGAGCGAAGTCGACCCGAGAGAAA
19	Meq-ZIP_del-F	AGACGGACTATGTAGACAAAGAGCCAGTTTGCCCTATGGC
20	Meq-ZIP_del-R	GCCATAGGGCAAACTGGCTCTTTGTCTACATAGTCCGTCT
21	Meq-BZIP_del-F	TTTCTCTCGGGTCGACTTCGGAGCCAGTTTGCCCTATGGC
22	Meq-BZIP_del-R	GCCATAGGGCAAACTGGCTCCGAAGTCGACCCGAGAGAAA
23	chHDAC1-FLAG-F	gatc**GCTAGC**atg***GACTACAAAGACGATGACGACAAG*** gcgctgacgcaggggac
24	chHDAC1-HA-F	gatc**GCTAGC**atg***TACCCATACGATGTTCCAGATTACGCT*** gcgctgacgcaggggac
25	chHDAC1-R	gatc**GCGGCCGC**ttaggtggattttgtctctt
26	chHDAC1-N-160-R	gatc**GCGGCCGC**ttagatagccaggacaatatcgt
27	chHDAC1-N-320-R	gatc**GCGGCCGC**ttacaaagccacagcagtctcat
28	chHDAC1-53-C-F	gatc**GCTAGC**atgatatatcgccctcacaaggcg
29	chHDAC1-81-C-F	gatc**GCTAGC**atgccagacaacatgtctgagta
30	chHDAC1-121-C-F	gatc**GCTAGC**atggctgtgaagctgaacaagca
31	chHDAC1-161-C-F	gatc**GCTAGC**atgttggagctcttaaagtatca
32	chHDAC1-321-C-F	gatc**GCTAGC**atggacactgagatcccaaatga
33	chHDAC1-FLAG-R	gatc**GCGGCCGC**tta***CTTGTCGTCATCGTCTTTGTAGTC*** ggtggattttgtctcttcct
34	chHDAC2-FLAG-F	gatc**GCTAGC**atg***GACTACAAAGACGATGACGACAAG*** gcgtacagtcagggcgg
35	chHDAC2-HA-F	gatc**GCTAGC**atg***TACCCATACGATGTTCCAGATTACGCT*** gcgtacagtcagggcgg
36	chHDAC2-R	gatc**GCGGCCGC**tcaaggattgctgagctgtt
37	chHDAC2-N-160-R	gatc**GCGGCCGC**tcaggcaagcacaatatcattga
38	chHDAC2-N-320-R	gatc**GCGGCCGC**tcaggcaacagcagtttcatatg
39	chHDAC2-54-C-F	gatc**GCTAGC**atgatttaccgaccccacaaagct
40	chHDAC2-81-C-F	gatc**GCTAGC**atgaggcctgacaatatgtctga
41	chHDAC2-121-C-F	gatc**GCTAGC**atgggggcggtaaaattgaacag
42	chHDAC2-FLAG-R	gatc**GCGGCCGC**tca***CTTGTCGTCATCGTCTTTGTAGTC*** aggattgctgagctgttc
43	chHDAC1_qPCR_F	GGATGAAGAAGAAGAAGATCC
44	chHDAC1_qPCR_R	GATAACTATGCACTGACAGG
45	chHDAC2_qPCR_F	AAGGTGGACGGCGAAATG
46	chHDAC2_qPCR_R	GATACGGTCCATGCCAAATAG
47	chGAPDH_qPCR_F	GTCAACGGATTTGGCCGTAT
48	chGAPDH_qPCR_R	CCACTTGGACTTTGCCAGAGA

* The sequences that highlighted in bold are restriction enzyme sites.
** The sequences that highlighted in bold italics are the tag sequences.
